# Axial Crushing Theory and Optimization of Lattice-Filled Multicellular Square Tubes

**DOI:** 10.3390/ma17061245

**Published:** 2024-03-08

**Authors:** Xiwu Zhou, Jingdong Liu, Weifeng Rong, Benying Wu

**Affiliations:** School of Transportation, Civil Engineering and Architecture, Foshan University, Foshan 528000, China; xiwuzhou@163.com (X.Z.); xrr5239@163.com (W.R.); wubenying163@163.com (B.W.)

**Keywords:** square lattice multicellular tubes, crashworthiness, theoretical model, multi-objective optimization

## Abstract

A lattice-filled multicellular square tube features a regular cross-sectional shape, good energy consumption, and good crashworthiness, which is suitable for the design of energy absorbers in various protection fields such as automobiles, aerospace, bridges, etc. Based on the super folding theory, two reference planes are set to refine the energy consumption zone of the super folding element in this study. The energy consumption calculation of convex panel stretching is involved, and the critical crushing force formula is introduced in this study. Meanwhile, the calculation method from a single-cell square tube to a multicellular thin-walled square tube is extended and the structural optimization is investigated, in which the NSGAII algorithm is used to obtain the Pareto front (*PF*) of the crashworthiness performance index of the square multicellular tubes, the Normal Boundary Intersection (NBI) method is adopted to select knee points, and the influence of different cross-sectional widths on the number, as well as the thickness, of cells are discussed. This study’s results indicate that the theoretical value is consistent with that obtained from the numerical simulation, meaning that the improved theoretical model can be applied to predict the crashworthiness of multicellular square cross-sectional tubes. Also, the optimization method and study results proposed in this study can provide a reference for the design of square lattice multicellular tubes.

## 1. Introduction

The frequent use of transportation has led to a gradual increase in transportation speed and distance requirements, as well as stricter energy efficiency and safety requirements for energy-consuming buffers for transportation vehicles. Additionally, the increase in traffic flow and transportation tonnage will greatly increase the probability and severity of vehicle and ship collision accidents with bridge piers. Therefore, impact protectors in bridge engineering also need to have higher energy consumption efficiency and impact resistance performance. Thin-walled metal structures are often used for buffering and energy consumption in various engineering fields. Due to their stable function, suitable buffering strength, and high-quality specific energy absorption, thin-walled metal structures can not only be used alone for simple engineering protection, but also can be used as a container or peripheral structure combined with many buffering materials to form a composite energy consumption system [1,2]. In civil engineering, thin-walled metal structures often play an important role in impact buffering at critical locations of bridges and buildings. For example, in the event of accidents like ship bridge collisions and vehicle column collisions, metal tube sleeves can provide effective impact protection [3,4]. Due to the stable axial force and good balance of the spatial shape of the tube, thin-walled metal structures are commonly used in engineering [5].

The manifestation of axial crushing of thin-walled metal tubes is relatively complex, wherein factors such as the shape of the tube section, the aspect ratio of the tube body, and the grid structure inside the tube can all have an impact on the bending mode and collision resistance of thin-walled tubes. So far, researchers have conducted intensive research on the crushing shapes and energy consumption of thin-walled tubes. The calculation of axial crushing force and energy consumption formula for thin-walled tubes can be generally divided into two aspects: an analytical method based on the law of energy conservation and the principle of minimum potential energy [6,7], and a numerical method obtained by fitting experimental conclusions and specifications with data [8]. In contrast, the folding model theory obtained via the analytical method is more accurate and applicable. With the widespread application of thin-walled metal tubes in engineering, researchers have deepened their theoretical research on the analytical solutions of folding elements, resulting in the improvement and perfection of the folding element model. The two common models in relation to the analytical method are the two-dimensional progressive folding model and the three-dimensional super folding element model. In a two-dimensional folding model, the stacked sections are analyzed, and geometric relationships and bending positions are highlighted [9]. Wierzbicki et al. [10] proposed a simplified folded-lobe model in their study of cellular structure, believing that the tube walls can ultimately close together. Mahmoudabadi et al. [11], who put forward a more accurate two-dimensional folding model, consider that the actual folding lobe of the cell wall does not fit well with each other. Yao et al. [12] proposed a three-hinge-line folding theory for thin plates based on static compression and the study of two-dimensional folding of cellular structures. On account of the folding deformation of thin-walled tube walls, which have a similar folding shape to thin plates, the three-hinge-line models can serve as a reference for analyzing the folding behavior of thin-walled tube walls in theoretical research, thereby simplifying theoretical calculations. The three-dimensional super folding element theoretical model is more specific and targeted at the crushing of square tubes in terms of calculation. Abramowicz [13] was the earliest to summarize the crushing modes of square tubes through a large number of experiments, which can be divided as follows: extended collapse mode, symmetric collapse mode, and asymmetric mixed collapse modes A and B. Meanwhile, two types of super folding elements (Type I and Type II) were proposed. In subsequent studies, Abramowicz, along with Bhat [14] and Wlodek [15], modified the superposed element theoretical model and applied it to the design of energy dissipators [16]. This folding model provides direction and a reference for the subsequent research and development of element folding theory. It can be found from the primary research that square tubes have many forms of crushing, especially upon actual impact, where they present great randomness [4]. However, the folding modes are mainly symmetrical collapses, and the tube wall shows concave and convex folding under the constraint of adjacent plates [17]. Early researchers such as Von Karman and Winter [18] reckoned that the stress distribution of thin plate sections in critical states is not uniform. They proposed the concept of an effective section method based on the characteristics of section stress. Shafer et al. [19] proposed the direct strength method based on the relationship between yield limit state and critical state, and calculated the critical stress of the entire section by introducing a reduction coefficient. The effective width method has also been adopted by relevant design codes for thin-walled steel structures in various countries due to its simplicity and practicality. The more-detailed calculation empirical formulas have been proposed in the specifications AISI in the United States and GB50018-2002 in China.

In the study of the axial crushing mechanical properties of multicellular tubes, Tran et al. [8,20] applied the super folding element theory into the crashworthiness design of multicellular thin-walled tubes and proposed a theoretical calculation formula for multicellular square tubes based on multiple combinations of angular elements, thereby enriching the calculation application of folding element theory in square multicellular tube structures. Zhang et al. [21] predicted the average force of a multicellular square thin-walled structure under axial compression with the number of cells, and verified the effectiveness of the model through the finite-element method. In addition, the simulation results show that when the number of cells is 3 × 3, the energy absorption efficiency can be increased by 50% compared to a single-cell tube. The above formula for the mean crushing force (*MCF*) of multicellular tubes is based on a simplified calculation model using corner elements, while Wang et al. [22] introduced the parameter of cell number based on the super folding element theoretical model, in which the predicted results of multicellular tubes are similar to the finite-element results, indicating that the super folding element theoretical model is also suitable for the calculation of multicellular tubes. In conclusion, there is still much room for improvement in the calculation of crushing force for multicellular square cross-sectional tubes. At present, multicellular tubes have more diverse spatial folding forms in terms of average force calculation, and the folding zones can be divided in detail. In terms of the calculation of the axial initial crushing force of multicellular tubes, although researchers pay less attention to it, it is equally important to obtain the critical formula for further improving the axial compression mechanical properties of multicellular tubes.

Accordingly, based on the influence of convex panel stretching on the energy consumption and axial collapse reaction of three-dimensional super folding elements, this study considers the characteristics of the cell wall folding lobe shape in the two-dimensional asymptotic model and proposes a two-reference-plane super folding element energy consumption calculation method for calculating energy consumption, while deriving the average force formulas. Meanwhile, the rationality of this folding theory is verified by using quasi-static compression experiments and finite-element simulation techniques. In terms of the peak crushing force, a peak force calculation formula based on the variation in the width–thickness ratio is proposed by referring to the American AISI and Chinese GB50018-2002 specifications. In addition, the expansion and optimization of the collapse theory of square multicellular tubes are discussed in detail. Among them, the optimized initial crushing force (*ICF*) and specific energy absorption (*SEA*) indicators are often obtained by constructing response surfaces. Additionally, the critical force expression for square multicellular tubes is derived, and the average force calculation formula and effective stroke simulation value are applied to the construction of the *SEA* response surface. Finally, an analysis of the optimization adaptation parameters of the number of cell pores and tube thickness from the perspective of maintaining the total cross-sectional width through optimization algorithms is made in this study, which seeks the optimal solution for square multicellular tubes of equal volume. As a result, this study can provide new optimization directions and effective references for engineering designs.

## 2. Experiments on Axial Compression

### 2.1. Preparation of Specimen

To verify the accuracy of the finite-element model, this study provides a reasonable reference for theoretical expansion. The single-cell square cross-sectional tubes were selected for axial compression, and 304 stainless-steel square tubes were selected as the experimental material. Moreover, considering that the material has no obvious yield stage, the stress value at a residual strain of 0.2% during stretching was selected as the yield strength. The relevant coefficients such as the Young’s modulus (*E*_0_), yield strength of the material (*σ*_0.2_), ultimate strength (*σ_u_*), and hardening coefficient (*n*) are listed in Table 1. Tubes with two thicknesses of 1.5 mm and 2.0 mm were selected for crushing tests, with cross-sectional sizes of 50 mm × 50 mm and 100 mm × 100 mm, respectively, and square tube heights of 150 mm.

### 2.2. Energy Consumption Indicators

(1) Initial crushing force (*ICF*) refers to the force that the tube bears under compression, when plastic deformation begins to occur. In the entire load–displacement curve, *ICF* generally manifests as the peak compressive force of the tube body.

(2) Mean crushing force (*MCF*) refers to the parameter that measures the average force on the tube body during the collapse of the square tube, which is obtained by calculating the rate of energy consumption before the square tube enters the compression-dense zone to the compression stroke:

(1) $$MCF=\frac{E_{e}}{\delta_{e}}.$$

**Figure 1:**
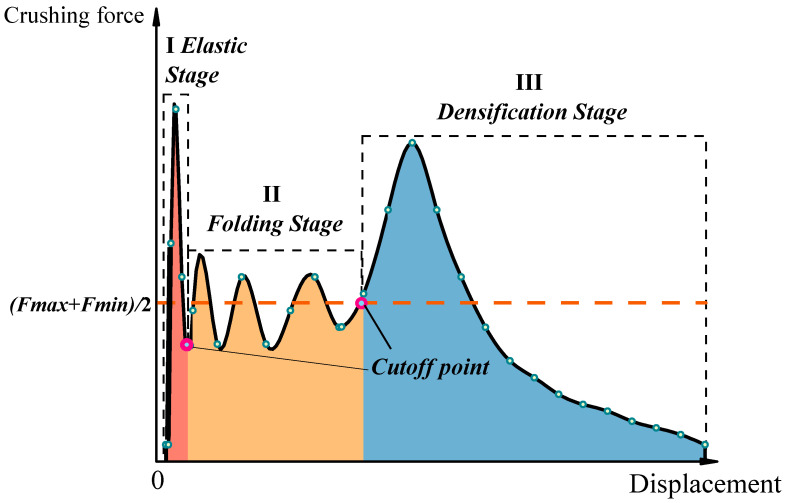
Schematic diagram of axial crushing load displacement of the tubes.

In this equation, *δ_e_* represents the effective compression stroke, and *E_e_* refers to the effective crushing energy consumption. To better distinguish the cut-off position between the folded section and the dense section, the average value of the maximum and minimum forces in the folding stage is selected as the reference point for the cut-off position, and searches for the closest value near the final trough of the collapse curve as the cut-off point to distinguish the folded section and the dense section, as shown in Figure 1.

(3) Crushing force efficiency (*CFE*) represents the degree of difference between the initial peak load and the average load during the compression process, which can be expressed as follows:

(2) $$CFE=\frac{MCF}{ICF}\times 100\%.$$

(4) Specific energy absorption (*SEA*):

(3) $$SEA=\frac{E_{e}}{m}.$$

### 2.3. Quasi-Static Compression Tests

During the experimental process, an electro-hydraulic servo universal testing machine is used to conduct quasi-static compression tests on the square tube specimens to determine the crushing force and displacement data, verifying the rationality of the improved calculation method for energy consumption and crushing force of the tube folding element. The compression rate is set to 0.5 mm/min. To ensure that the tube wall can rotate freely as much as possible, Vaseline is applied to the contact surface of the pressure head or support of the tubes. In addition, square tubes with the height of 150 mm for the section sizes of 100 mm × 100 mm and 50 mm × 50 mm are selected, respectively. In addition, the relevant test specimen size parameters and test results are presented in Table 2. According to the set size of the square tube, for T1 and T2, an element width of *c* = 25 mm was selected, and for T3 and T4, a width of *c* = 50 mm was selected. The data summary after comparing the *ICF* and *MCF* obtained from the experiment with the theoretical calculation values is shown in Table 2. For comparison, the experimental results and figures are shown in figure in Section 3.2.

According to the experiment, compared to the width of the square tube, the change in wall thickness has a greater influence on the average folding force and initial peak folding force, indicating that thin-walled structures have sensitivity to thickness.

## 3. Verification of the Finite-Element Model

### 3.1. Model Parameters

**Figure 2:**
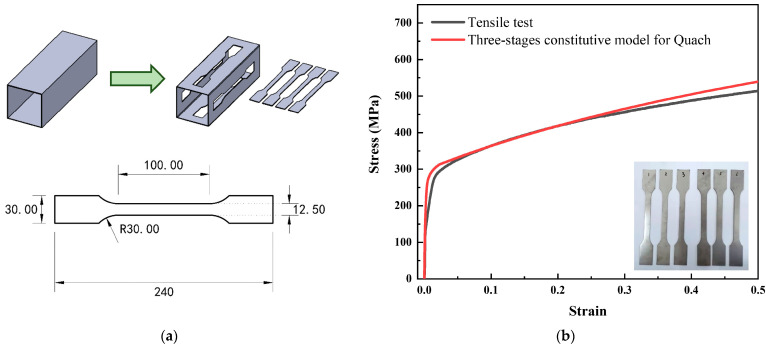
Tensile test and results of the specimens: (**a**) size of specimens (mm); (**b**) stress–strain curve of the stainless steel material.

In order to further verify that the energy consumption and crushing force calculated by using the improved folding element method were consistent with the results of the tube collapse, finite-element software ABAQUS (Software version 6.14) was used to establish the tube model and conduct a static loading simulation in this study. Quach [23] modified the Ramberg Osgood stainless-steel constitutive model [24], in which the improved stainless-steel constitutive model was more consistent with the experimental results. In this study, tensile specimens were cut from the stainless-steel square tube surface to reduce experimental errors, with the dimensions of the specimens meeting the requirements of ASTM E8M specification [25], as shown in Figure 2. The tensile stress–strain curve is obtained by using a microcomputer-controlled electro-hydraulic servo universal testing machine to conduct quasi-static tensile tests on the specimens. Comparing the Quach constitutive curve with the experimental curve, as shown in Figure 2, it is obvious that the yield strength and stress growth trend of the material show good agreement. Therefore, the three-stage stress–strain model from Quach in this study was selected as the input for the constitutive parameters of 304 stainless steel to the ABAQUS*Material_PLASTIC module.

**Figure 3:**
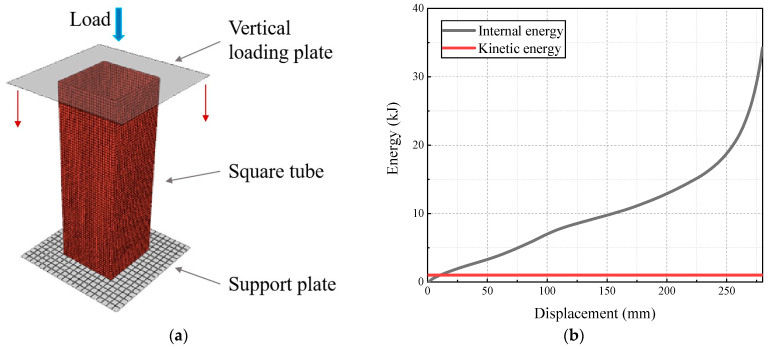
Finite-element model of the square tubes: (**a**) schematic diagram of FE model; (**b**) relationship between internal energy and kinetic energy.

The finite-element modeling and mesh division of the square tubes are shown in Figure 3a, where the upper and lower ends of the tube are, respectively, loaded, and their contact mode is defined as general contact with a contact friction coefficient of 0.15. The lower bearing plate is defined as fully fixed, while the upper loading plate is a discrete rigid body with only longitudinal displacement. Considering the loading condition of the actual test, Smooth Step is adopted to control the displacement loading process. In this study, three-dimensional shell element S4R was selected as the type of tube element. The finite-element model should ensure the accuracy while improving the computational efficiency. Rough meshes will bring the problem of calculation deviation, while over-dense meshes will lead to excessive calculation time cost and calculation errors. After comparing the size in the model and referring to the literature [7,26], the mesh sizes were defined to be less than 2 mm × 2 mm and the number of meshes was controlled to be about 19,200. To improve the calculation efficiency, the loading speed is amplified to 100 mm/s: at this time, the relationship between the overall kinetic energy and internal energy of the model is shown in Figure 3b, and the ratio of kinetic energy to internal energy is not greater than 5%, indicating that the model ignores the influence of kinetic energy.

### 3.2. Model Validation

**Figure 4:**
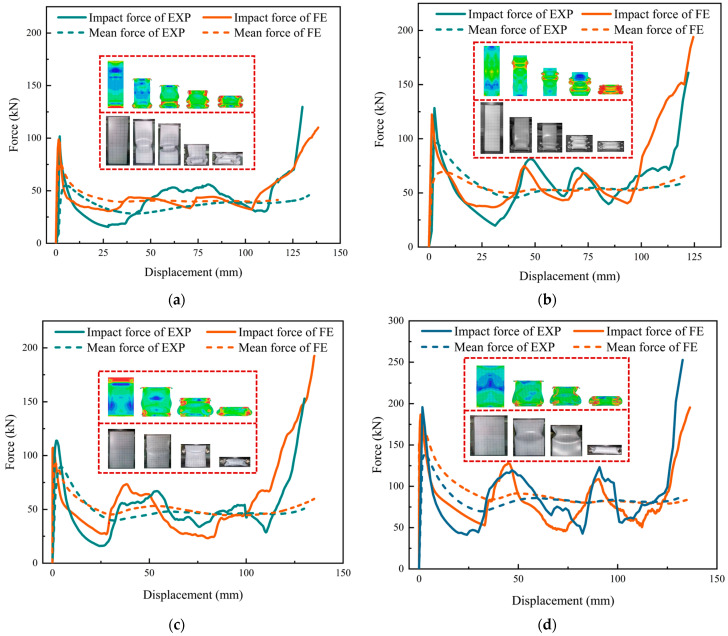
Simulation and test results: (**a**) T1; (**b**) T2; (**c**) T3; (**d**) T4.

To verify the correctness of the finite-element parameter values and the suitability of the constitutive model and loading method, the axial compression experimental data of the square cross-sectional tubes in Section 2.3 were compared with the compression data of the corresponding finite-element model of the cross section parameters, which are shown in Figure 4 and Table 2.

The load–displacement curves in Figure 4 obtained from the test and FE simulation have similar variation trends and can correspond to the folding position of the square tubes. As can be seen in Table 2, the errors between the test results and the FE data are within 6%, and the maximum difference is 8.92 kN. In general, the *ICF* obtained through the experiment presents a larger value than that obtained from the finite-element model, which means that the error fluctuation of the average value is relatively large. On the whole, the FE simulation is more accurate in terms of *ICF* and *MCF*, so the basic parameters of the finite-element model in this section are also used for the model setting in the numerical simulation discussion process in the following sections.

## 4. Improved Folding Model for Corner Elements

### 4.1. Corner Element Theory Considering Convex Panel Stretching

**Figure 5:**
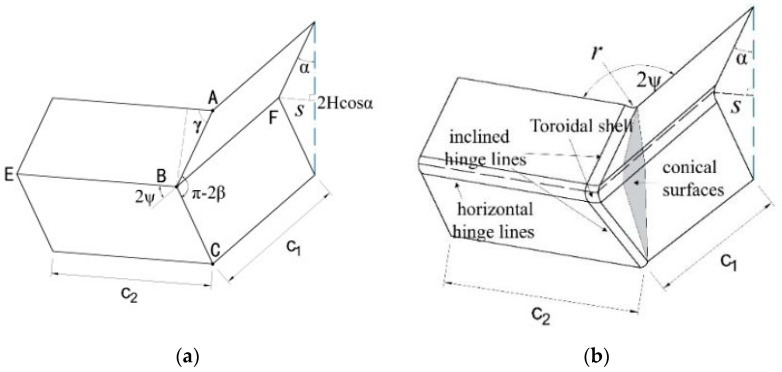
Folding element model from Abramowicz: (**a**) classic super folding element [6]; (**b**) expanded super folding element [15].

The four angles of a square tube are typical corner elements. During the compression process, when the axial load is relatively stable, the tube is prone to exhibit symmetrical folding modes. It can be found from the literature that the folding mode of obtuse-angle elements is mainly Type II, while the folding mode of acute-angle and right-angle elements is mainly Type I [15]. The element angles discussed in this study are all right angles, so the super folding element Type I model of thin-walled square tubes during axial compression deformation is used as the calculation prototype to analyze and improve the axial compression mechanical behavior of the tubes under compression loads. This super folding element consists of four trapezoidal surfaces and one toroidal shell element, with each folding element representing 1/4 of the tube section at element height. The parameters of the folding element are shown in Figure 5a.

**Figure 6:**
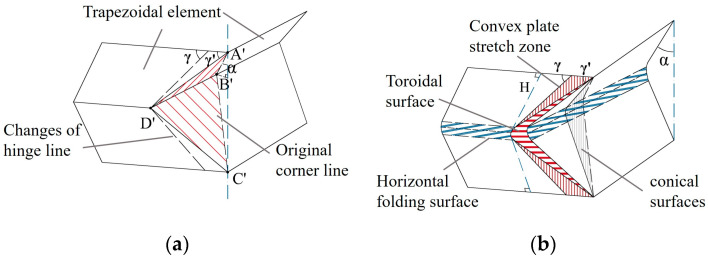
Super folding element considering convex panel stretching: (**a**) changes in the migration zone and oblique hinge line; (**b**) schematic diagram of element model. (Note: The slash lines represent the folding positions).

The extended super folding element model proposed by Abramowicz [15] takes into consideration the energy consumption effect of the conical surface area formed during the folding process, which is considered to be obtained through the tensile deformation of the tube wall during the folding process. The tube corner lines in this model are flattened, forming an oblique traveling hinge line on the outer convex of the tube wall and causing movement (Figure 5b). In fact, the element rotates in two directions during the folding process. Furthermore, the convex trapezoidal panel of the element is not only formed during the inclined hinge movement, but also exhibits local stretching during the folding process in both directions. This stretching area is called the convex stretching area, and this folding form of the element is shown in Figure 6. After the stretching of the convex surface, new energy consumption zones and larger annular shell surfaces occur (Figure 6a,b). Therefore, an intensive study of the folding energy consumption of the element is needed in the future.

**Figure 7:**
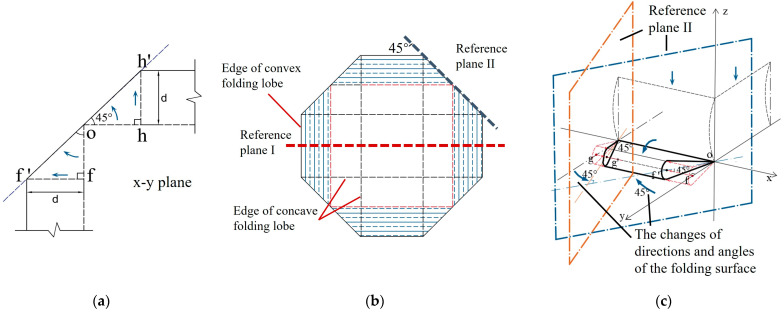
Deformation diagram of square tube folding: (**a**) local display of folded lobes; (**b**) vertical view of the box-shaped tubes after crushing; (**c**) a droplet-shaped wedge with oblique section.

From the comparison of the respective shapes before and after the axial compression of the square tube, it is found that the super folding element forms a symmetrical curved folded lobe after compression. The stretching of the convex plane is considered in the improved folding element, and the movement of the folded lobe on the edge line of the plane is shown in Figure 7a. Based on experiments and simulations, it can be seen that when a single element is in a fully crushing state, the angle between the tube walls changes from 2*ψ*_0_ = 90° to a fully unfolded angle of 180°. Assuming that the tube angle is symmetrically unfolded in both directions of the tube wall, the cross section of the tube crushing is octagonal, with an angle of 45° between the oblique edge and the horizontal line [7] (Figure 7b), in which the folded lobe forms a wedge-shaped structure with a water droplet-shaped oblique section (Figure 7c). Generally, changes in the edge lines of the element will affect the energy consumption calculation of the annular shell surface. Given the computational requirements, the element model is divided into five categories of zones in this study, and in the following sections, a comprehensive analysis of the energy consumption calculation methods for these five improved deformation zones is made. The five types of deformation regions are divided into sections based on the deformation characteristics of the model, as shown in Table 3.

**Figure 8:**
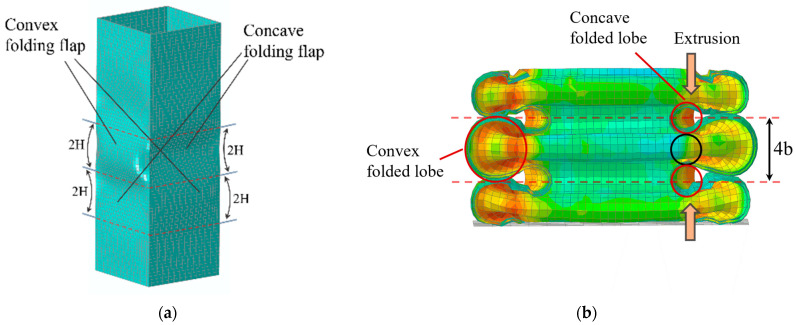
Spatial folded-lobe model: (**a**) initial buckling of square tubes; (**b**) complete formation of folded lobe.

**Figure 9:**
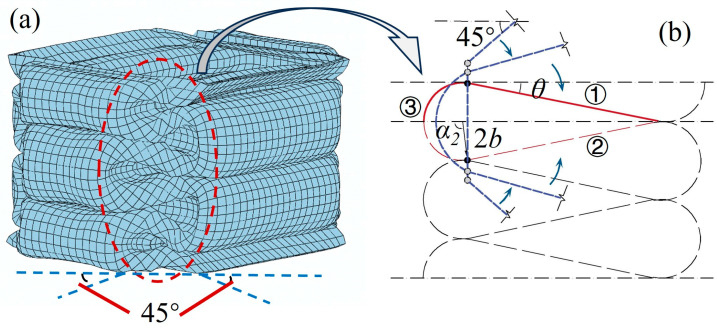
Schematic diagram of reference plane II and deformation of double-hinged-line model: (**a**) space stacking; (**b**) model of the double-hinged line.

(1) Reference plane I: The deformation of the folded lobe can be clearly observed through the projection of the square tube on reference plane I. In the classical folding element theory, the projection plane of the folded lobe on plane I is completely closed on the wall of a single tube [6,8,12]. In fact, the folded lobe angle α is not strictly equal to 90°, and the deformation of the inner and outer tube walls during the folding process is slightly different. Malekshahi et al. [7] proposed that there is a 1:2 rate relationship between the bending radius of the concave valve and the convex valve in the box-shaped tubes, which will result in a change in the calculation formula for the height and radius of the folded element. From this model, it can be observed that the element height and deformation amplitude are the same in the initial stage of the formation of the outer convex surface and the inner concave surface (as shown in Figure 8a). Under axial load, the inner concave and outer convex surfaces continue to compress with each other after contact, and the bending radius of the inner concave lobe is reduced until the same compression segment as the outer convex lobe is formed. At this time, the bending radius of the outer convex lobe is exactly twice that of the inner concave lobe (Figure 8b). Therefore, the outer folded lobe and the inner folded lobe have the same element height, but the reason for their different bending radii after complete collapse is due to differences in the degree of compression. From what has been discussed above, the folding theory based on the oblique traveling-hinge region and the horizontal fixed-hinge zone of reference plane I are divided into two parts for discussion: the inner folded lobe and the outer folded lobe.

(2) Reference plane II: For the convenience of model calculation, the cellular thin-walled folded-lobe model proposed by Mahmoudabadi [11] is used as a reference in this study, and the water droplet-shaped folded line on the reference plane II is equated to a double-hinged line. Additionally, the simplified diagram is shown in Figure 9a,b, in which the hinge lines of **①** and **②** in the Figure 9b model exhibit deflection, while the hinge line **③** shows arc-shaped bending. Considering that the folding line on the inclined plane II is formed from the edge of the outer folded lobe, the radius of the circular arc in the model is the same as the bending radius of the folded lobe. Moreover, *θ* is the angle between the trapezoidal panel and the horizontal direction after the element is folded, and *α*_2_ is the final inclined angle of the folded lobe in Figure 8. In addition, the length of the double-hinged line is changed into $2\sqrt{2}H$ because of the concave deformation occurring inside the tube.

### 4.2. Folded-Lobe Model on Reference Plane I

The energy consumption calculation of the horizontal fixed hinge, oblique traveling hinge, and conical surface stretching on reference plane I is the same as the calculation method of the classical super folding element. However, separate discussions are needed during calculation due to the difference in bending radius between the concave and convex lobes. The rotation of the horizontal fixed-hinge element is related to the rotation angle of the longitudinal element. Furthermore, the radius of the inner folding is set as *b*, the radius of the outer folding is set as 2*b*, and the final rotation angle is set as *α*_1_. Meanwhile, the flow stress *σ*_0_ of the material is taken as the equivalent stress during element deformation. For metal materials with a hardening index of *n*, and $\sigma_{0}=\sqrt{\frac{\sigma_{y}\sigma_{u}}{1+n}}$, then $M_{0}=\frac{\sigma_{0}h^{2}}{4}$ can be obtained. The average value of element width is set as $c_{m}=\frac{\left(c_{1}+c_{2}\right)}{2}$, and the energy consumption calculation of the horizontal fixed hinge can be written as follows:

(4) $$W_{H}=\sum_{2}^{i=1}\int_{0}^{\alpha_{1}}c_{i}M_{0}d\alpha =2M_{0}c_{m}\alpha_{1}.$$

The length of the oblique traveling hinge is set as *L*; then, $L=\frac{2H}{\sin\gamma }$. From movement characteristics of the oblique traveling hinge, it can be inferred that the folding degree changes with the distance from the moving point to the horizontal fixed hinge. Additionally, the bending radius of the hinge is set as *b*. According to the energy consumption calculation method of the traveling hinge, the energy consumption rate of the oblique traveling hinge is

(5) $$\overset{•}{W_{L}}=2M_{0}L\frac{v_{t}}{b}.$$

Integral calculations are performed in α on both sides of Equation (5), and the energy consumption calculation of the oblique traveling hinge can be written as follows:

(6) $$W_{L}=\frac{4M_{0}H^{2}}{b\tan\psi_{0}}\int \frac{\cos\alpha }{\sin\gamma }d\alpha .$$

From the geometric relationships in the folding element model, $\tan\gamma =\frac{\tan\psi_{0}}{\sin\alpha }$, for α ∈ [0, $\alpha_{1}$], making $I_{L}=\frac{1}{\tan\psi_{0}}\int_{0}^{\alpha_{1}}\frac{\cos\alpha }{\sin\gamma }d\alpha$, we finally can obtain the following:

(7) $$W_{L}=\frac{4M_{0}H^{2}I_{L}(\psi_{0})}{b}.$$

The conical zone changes with the folding angle, and according to Abramowicz’s research [16], the energy consumption calculation of the conical surface stretching can be written as follows:

(8) $$W_{C}=\int_{A}\frac{2M_{0}}{b}dA=\frac{4M_{0}H^{2}I_{C}(\psi_{0})}{h},$$

where $I_{C}=\int_{0}^{\alpha_{1}}\frac{\cos\alpha \tan(\psi_{0})}{1+\tan^{2}(\psi_{0})\sin\alpha }d\alpha$, and *A* is the total area under the oblique traveling hinge of the super folding element.

### 4.3. Double-Hinged-Line Model on Reference Plane II

**Figure 10:**
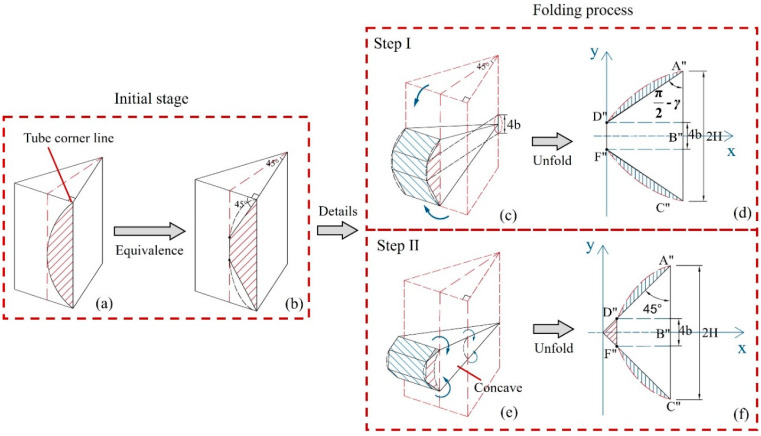
Movement process and equivalent schematic diagram of the traveling hinge of the folding element: (**a**) the initial tube angle and the zone to be moved; (**b**) equivalent zone to be moved; (**c**) folding diagram of Step I; (**d**) equivalent movement zone of Step I; (**e**) folding diagram of Step II; (**f**) equivalent movement zone of Step II.

On reference plane II, the stretching of the convex panel and the toroidal shell surface is related to the movement features of the oblique traveling hinge and the folding angle of the trapezoidal plate. The traveling hinge moves from the concave to the convex surfaces, and a sweeping concave shape occurs in the tube along the corners of both ends of the element. In this way, an arched area is formed, as shown in Figure 10a. The folding process of the movement zone includes two steps, in which Step I is the angle α_1_ that occurs on reference plane I (Figure 10c,d), and Step II is the angle α_2_ that occurs on reference plane II. Finally, the traveling hinge is fixed at a position with an angle of 45° to the edge of the panel (Figure 10e,f). According to the simplified model of the double-hinged line, the arch curve is equated to an equilateral trapezoidal curve, as shown in Figure 10b, and the folded zone forms a trapezoidal zone after being fully unfolded, as shown in Figure 10f. In order to compensate for the area differences between the equivalent zone and the arched zone, the equivalent trapezoidal zone of the double-hinged-line model is expanded into a triangular zone in this study.

#### 4.3.1. Stretching of Convex Panels

**Figure 11:**
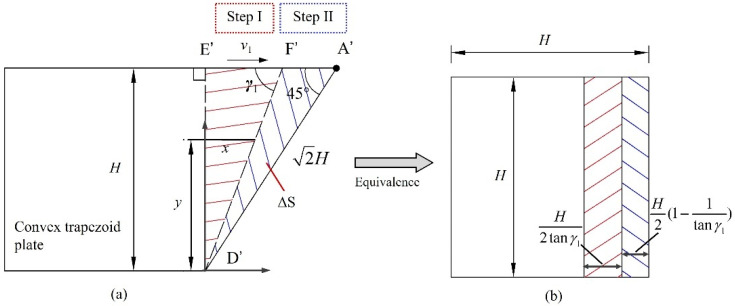
Deformation of convex trapezoidal panels: (**a**) stretching area; (**b**) stretching equivalence.

Although both Step I and Step II occur simultaneously during the folding process, there is considered to be no coupling effect between Step I and Step II. Therefore, it is assumed that the stretching of the trapezoidal plate surface only occurs in Step II during analysis. In addition, the angle between the sweeping stopping line of Step I and the horizontal edge of the element is set to be *γ*_1_, in which the convex panel is assumed to exhibit local deformation in the triangular area enclosed by the sharp angle *γ*_1_, the oblique traveling hinge line A′D′, as well as the right-angle edge E′D′, as shown in Figure 11a. Since *γ*_1_ is a determined value of *α*_1_, and the horizontal strain changes linearly with the change in *H*, the energy consumption calculation can be simplified by area. Additionally, the triangular area on the convex surface is equated to the area, as shown in Figure 11b; then, the total energy consumption of the convex panel stretching based on geometric relationships can be written as follows:

(9) $$∆S=\frac{H^{2}}{2}(1-\frac{1}{\tan\gamma_{1}}),$$

(10) $$W_{p1}=\sigma_{0}h∆S.$$

Based on Equations (9) and (10), the total energy consumption of two convex panels stretching on an element can be written as

(11) $$W_{p}=\frac{4M_{0}H^{2}I_{p}(\psi_{0})}{h},$$

where $I_{p}(\psi_{0})=\frac{\sin\alpha_{1}-\tan\psi_{0}}{\sin\alpha_{1}}$.

#### 4.3.2. Stretching of Toroidal Shell Surface

**Figure 12:**
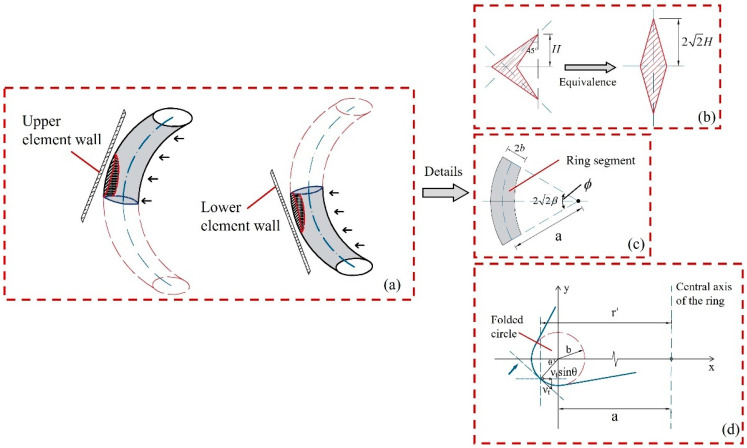
Schematic diagram of a circular segment passing diagonally through the tube wall: (**a**) through the element surface; (**b**) equivalent area of toroidal shell; (**c**) circular segment; (**d**) contact between element surface and circular segment cross section.

The formation of creases on a curved surface and the movement of oblique traveling hinges occur simultaneously in time and interact with each other. The folding element exhibits concavity in the surface, resulting in the generation of traveling hinges, and the surface of the toroidal shell also changes with the change in traveling hinges. In super folding theory, the formation of a toroidal shell surface can be considered as the plastic deformation generated when a toroidal surface passes through the thin plate [27]. For square thin-walled tubes, the crushing of the tube will ultimately form deformation outside the surface with the tube corner line as the axis of symmetry. When calculating the stretching energy consumption of the toroidal shell surface, the shape of the toroidal shell surface is simplified to a symmetrical extension with an oblique traveling hinge as the central axis. In this study, the calculation method for stretching energy consumption of the toroidal shell surface in the super folding element is adopted, and great importance is attached to the variation in the stretching surface. The shell surface is regarded as two identical circular bodies diagonally passing through the upper and lower trapezoidal tube surfaces of the folding element, as shown in Figure 12a. The stretching area of the toroidal shell is approximate to the area of the quadrilateral-shaped arrow in Figure 12b. For calculation, the arrow shape is further equivalent to a diamond shape. According to the super folding theory, the equivalent area needs to meet the following requirements: (1) If the central axis of the approximate graph develops along a 45° angle with the edge of the element, the height of the equivalent diamond is $2\sqrt{2}H$; (2) The stretching width of the toroidal shell surface increases linearly with the change in the contact arc *ϕ* of the circular segment; (3) Tensile strain occurs on the toroidal shell surface, which ignores the bending strain generated during the formation of creases.

The schematic diagram of the circular segment and the parameters of the circular segment are shown in Figure 12c,d, where the ring width of the circular segment is 2*b*. The distance between the center of the circular section and the axis of rotation of the ring body is $\sqrt{2}a$. The distance from the point on the shell surface to the center of the circular segment is *r*′. Based on the folding theory and the requirement (1), satisfied by the equivalent zone, the maximum value of the contact arc *ϕ* of the equivalent circular segment is $\sqrt{2}$*β*, namely, $\phi \in [-\sqrt{2}\beta ,\sqrt{2}\beta ]$. The contact arc *θ* between the cross section of the circular segment and the tube surface needs to meet the requirement of $\theta =[\frac{\pi }{2}-\psi_{0},\frac{\pi }{2}+\psi_{0}]$, in which the tangential velocity of the circular segment passing through the tube surface is $v_{t}=\frac{H\cos\alpha d\alpha }{\tan\psi_{0}}$, and the circumferential strain rate is $\overset{•}{\varepsilon_{\phi }}=\frac{v_{2}\sin\theta }{r\prime }$. From the geometric relationship, the following expressions can be obtained as follows:

(12) $$r\prime =b\cos\theta +\sqrt{2}a,$$

(13) $$\beta =\arctan\frac{\tan\alpha }{\sin\psi_{0}}.$$

Given the requirements satisfied by the equivalent zone, the curvature of the toroidal shell surface stretching varies linearly with the change in *ϕ*; then, $\psi =\psi_{0}+\frac{\pi -2\psi_{0}}{\pi }\phi$ can be obtained. During the formation process of the ring shell surface, the energy consumption of bending microstrain is the infinitesimal of higher order of energy consumption of tensile strain, which can be ignored in calculation [6]. The ultimate membrane force at the yield of the tube wall is set as *N*_0_, and the expression for the plastic energy consumption rate of the improved toroidal shell surface element when considering tensile strain can be written as

(14) $${\overset{•}{W}}_{S}=2\int_{0}^{\sqrt{2}\beta }\int_{\frac{\pi }{2}-\psi }^{\frac{\pi }{2}+\psi }N_{0}{\overset{•}{\varepsilon }}_{\phi }bd\theta r\prime d\phi .$$

Specifically, combined with Equations (13) and (14), integral calculations are performed in *θ* and *ϕ* on both sides of the equation, respectively; then, the relationship between the plastic energy consumption rate of the toroidal shell surface and the longitudinal tube surface rotation angle is obtained, which can be detailed as

(15) $$\overset{•}{W_{S}}=\frac{4N_{0}bH\pi \cos\alpha \overset{•}{\alpha }}{(\pi -2\psi_{0})\tan\psi_{0}}[\cos\psi_{0}-\cos(\psi_{0}+\sqrt{2}\beta \frac{\pi -2\psi_{0}}{\pi })].$$

where both $\overset{•}{W_{S}}$ and $\overset{•}{\alpha }$ are differentials with respect to time t. Combined with Equation (15), integral calculation is performed in α on both sides of the equation, and the energy dissipation can be obtained by the following:

(16) $$W_{S}(\alpha )=\frac{16M_{0}bHI_{S}(\psi_{0})}{h},$$

where $I_{S}(\psi_{0})=\frac{\pi }{(\pi -2\psi_{0})\tan\psi_{0}}\int_{0}^{\alpha_{2}}\cos\alpha \{\cos\psi_{0}-\cos[\psi_{0}+\frac{\sqrt{2}(\pi -2\psi_{0})}{\pi }\arctan\frac{\tan\alpha }{\sin\psi_{0}}]\}d\alpha$.

## 5. Formula for Folding of Square Lattice Multicellular Tubes

### 5.1. Calculation Model

**Figure 13:**
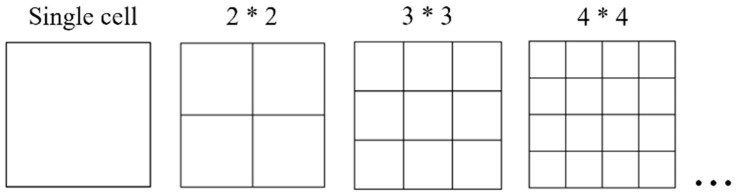
Square lattice multicellular tubes.

**Figure 14:**
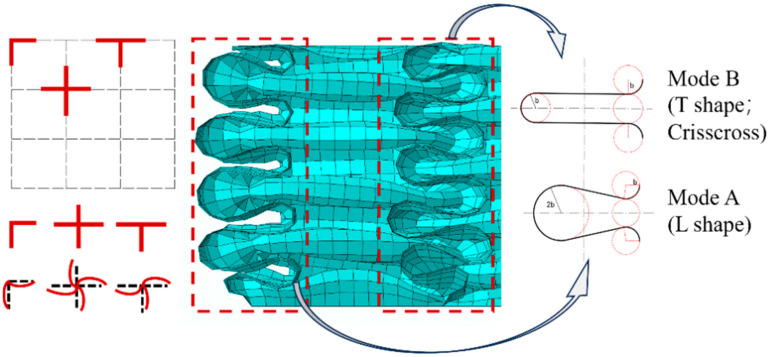
Inner and outer folded lobes of L-shaped, T-shaped, and crisscross-shaped elements (Mode A; Mode B).

Unlike single-cell square tubes, the internal folding form of square lattice multicellular tubes (Figure 13) is more complex. The elements can be divided into three types based on the number of folded lobes formed by the same connection points and the differences in folded deformation: namely, L-shaped, T-shaped, and crisscross elements [8,28]. The folding elements of simple single-cell square tubes pertain to the L-shaped element, which has large outer folded lobes and small inner folded lobes. The folded-lobe model under L-shaped elements is defined as Mode A. From these three types of folding lobes (Figure 14), it is apparent that the folded lobes on both sides of the T-shaped and crisscross elements, which are compressed by adjacent invaginated tube walls, occur in regular and compact stacking shapes. Moreover, the folding amplitude and radius of two types of folded lobes on both ends are the same, which is similar to the inner folded lobe in Mode A and was mentioned in Malekshahi’s research [28]. But this mode has not been applied to the calculation of multiple-shaped element types in multicellular tubes. Consequently, the folded-lobe model that is different from the L-shaped element is defined as Mode B.

The size of the two lobes in Mode B is equal, so the folding angle changes to α_1*B*_ = 90°, which is the same as the folding calculation angle of the classical super folding model, indicating that the folding angle in the classical model is relatively large for the calculation of L-shaped elements. The respective energy consumption of the horizontal fixed hinge and the oblique traveling hinge corresponding to the model after being compressed at both ends are demonstrated as follows:

(17) $$W_{HB}=2\pi M_{0}c_{m},$$

(18) $$W_{LB}=\frac{4M_{0}H^{2}}{b\tan\psi_{0}}\int_{0}^{\alpha_{1B}}\frac{\cos\alpha }{\sin\gamma }d\alpha =\frac{4M_{0}H^{2}I_{LB}(\psi_{0})}{b}.$$

### 5.2. Number of Quantitative Parameters on Areas Divided by Element Type

In a square lattice multicellular cross-sectional tube, the proportion of energy consumption in the L-shaped, T-shaped, and crisscross elements for the five energy consumption zones is not entirely the same. Zhou [29] quantitatively classified each type of folding and described their respective energy consumption calculation formulas, and suggested that the constraint coupling effect of adjacent tube walls can be ignored when calculating energy consumption and average force. The energy consumption areas of T-shaped and crisscross tube walls can be overlapped. For the improved folding element, the quantitative description of the five models is shown in Table 4. In Section 4 and Section 5.1, the energy consumption formulas for the L-shaped, T-shaped, and crisscross elements are discussed, respectively.

For square tubes with a square cross section, the spacing and number of cells on adjacent tube walls are equal. Assuming that each edge contains N cells, the number of L-shaped, T-shaped, and crisscross elements in the tube can be expressed as follows:

$$N_{L}=4;$$

$$N_{T}=4(N-1)$$

$$N_{C}={\left(N-1\right)}^{2}.$$

The L-shaped element is the most basic element in a square cross-sectional tube, occurring only at the four corners of the tube, which pertains to the folded lobe of Mode A, and can be directly calculated by using the folded-lobe model in Section 4. The T-shaped element occurs in the middle of the tube wall, which is the connecting part between the tube wall and the internal lattice. The flange part of the T-shaped element pertains to the folded lobe of Mode A. However, the inner wall of the T-shaped element is compressed by the flanges on both sides during crushing, so the inner wall of the T-shaped element pertains to the folded lobe of Mode B. The crisscross element occurs at the intersection of the square lattice inside, and the edges of each element are compressed against the lattice walls on both sides, so the crisscross element belongs to the folded lobe of Mode B. Supposing that each folded lobe corresponds to a model, the number of folded-lobe models and double-hinged-line models in the *N* × *N* lattice-filled multicellular cross-sectional square tubes are summarized in Table 5.

### 5.3. Integration of Element Energy Consumption

The energy consumption formulas are discussed separately based on the number of zones and the number of folded-lobe models for the three types of elements, in which the L-shaped element is applicable to the right-angle folding elements in Section 4. To distinguish the angles, the angles of the folded lobes of Mode A and Mode B on reference plane I are set to α*_1A_* and α*_1B_*, respectively. In view of Equations (5), (6), and (18), as well as Table 5, it is obvious that only four L-shaped elements occur in any multicellular square tube. Therefore, the calculation formula of energy consumption for the L-shaped super folding element of the multicellular square tube can be detailed as

(19) $$E_{L-\mathrm{shape}}=16M_{0}\left[c_{m}\alpha_{1A}+\frac{H^{2}}{b}I_{LA}(\frac{\pi }{4})+\frac{H^{2}}{h}\left(I_{CA}(\frac{\pi }{4})+I_{p}(\frac{\pi }{4})\right)+\frac{4Hb}{h}I_{S}(\frac{\pi }{4})\right].$$

where $I_{LA}(\frac{\pi }{4})=\int_{0}^{\alpha_{1A}}\frac{\cos\alpha }{\sin\gamma }d\alpha$ is the relevant energy consumption coefficient of the oblique traveling hinge under Mode B, and *c_m_* is the average width of the cell.

The T-shaped element has the characteristics of both the folded lobes of Mode A and Mode B, in which the energy calculations for two modes are distinguished and integrated separately from the folding angle and mode. Specifically, the oblique traveling hinge is related to the flange of the T-shaped element and occurs in the middle of the two lobes, so the oblique traveling hinge follows the folding angle of α_1*A*_ in Mode B when moving in the axial direction, with the quantity of 4(N−1). From Table 4 and Table 5, the calculation formula of energy consumption for the T-shaped element can be demonstrated as follows:

(20) $$E_{T-\mathrm{shape}}=8M_{0}(N-1)\left[\begin{array}{l} 2c_{m}\alpha_{1A}+c_{m}\alpha_{1B}+\frac{2H^{2}}{b}\left(I_{LA}(\frac{\pi }{4})+I_{LB}(\frac{\pi }{4})\right)\\ +\frac{2H^{2}}{h}\left(I_{CA}(\frac{\pi }{4})+I_{CB}(\frac{\pi }{4})+I_{p}(\frac{\pi }{4})\right)+\frac{16Hb}{h}I_{S}(\frac{\pi }{4}) \end{array}\right].$$

The crisscross element can be divided into two right-angle elements for energy consumption calculation, but the folded lobes of the crisscross element only contain the characteristics of Mode B. From Table 4 and Table 5, this calculation formula can be written as

(21) $$E_{\mathrm{crisscross}}=8M_{0}{\left(N-1\right)}^{2}\left[c_{m}\alpha_{1B}+\frac{H^{2}}{b}I_{LB}(\frac{\pi }{4})+\frac{H^{2}}{h}\left(I_{CB}(\frac{\pi }{4})+I_{p}(\frac{\pi }{4})\right)+\frac{4Hb}{h}I_{S}(\frac{\pi }{4})\right].$$

### 5.4. Solution of Folding Angle and Average Force

**Figure 15:**
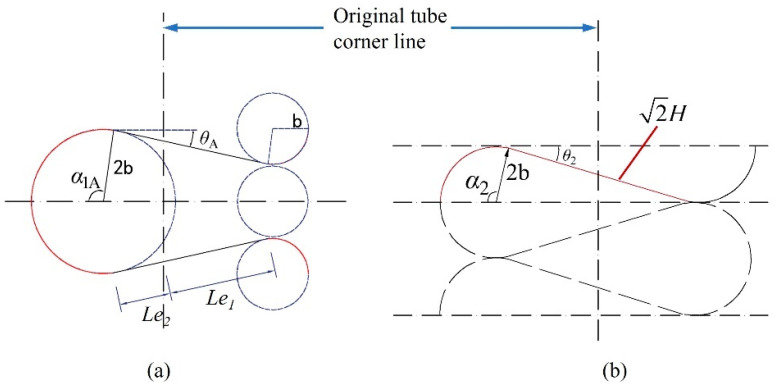
Schematic diagram and angle relationship of the cross section of folded lobes: (**a**) Mode A; (**b**) double-hinged-line model.

Due to the symmetry of a double-hinged-line model at the tube corner line, the folding angle is not affected by factors such as *c* and *h* (Figure 15b). Mahmoudabadi et al. [11] simplified the relation of α_2_ and *H*/*b*, specified the value of *H*/*b*, and obtained the value of α_2_ to be 1.7233. α_1*A*_ is greatly affected by the changes in *H*/*b*, which is set to $\alpha_{1A}=\frac{\pi }{2}+\theta_{A}$. The plane view of the folded-lobe model obtained with reference plane I as the cross section is shown in Figure 15a, which ignores the thickness of the tube wall. The relationships between effective length and *H*, effective length and *b*, angle and *H*, as well as angle and *b* are obtained from geometric relationships, which can be detailed as follows:

(22) $$Le_{1}=Le_{2}+b\alpha_{1}=2H-b\alpha_{1}.$$

(23) $$Le_{2}\sin\theta_{A}=\frac{Le_{2}}{Le_{1}+Le_{2}}b.$$

From Equations (22) and (23), the relation of *θ_A_* and *H/b* can be expressed as

(24) $$\sin\theta_{A}(2\frac{H}{b}-\frac{3\pi }{2}+3\theta_{A})-1=0.$$

**Figure 16:**
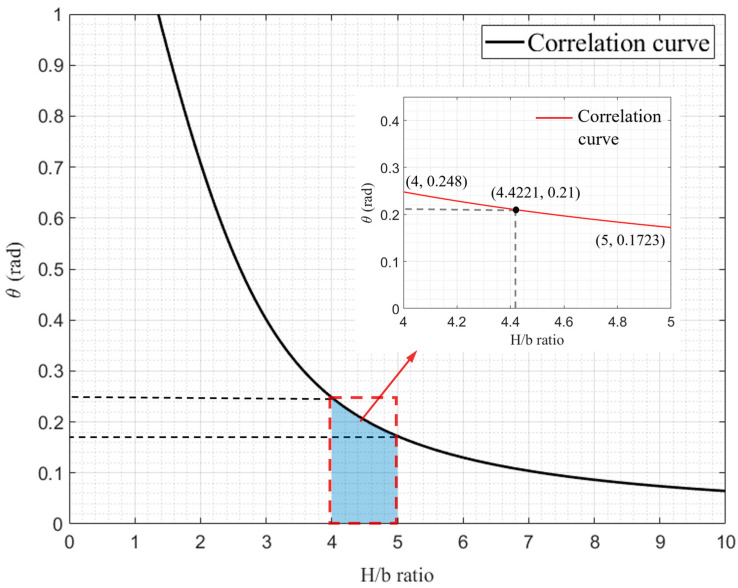
Details on the curve of *θ*-*H*/*b*.

The relation curve of $\theta_{A}-\frac{H}{b}$ is shown in Figure 16. As can be seen in Figure 16, the value of *θ_A_* is relatively small and the value of *H/b* in previous studies, such as in [7,11,18,30], is within the range of [4,5], so the corresponding range of *θ_A_* is [0.172, 0.248], and the change range in α_1*A*_ is within 4.34%. Malekshahi [7] defined the value of *θ_A_* as 0.21, which is within the range mentioned above. Considering that the values of *θ_A_*, *H*, and *b* are all unknown, it is more complex to solve for accurate values, while the range of *θ_A_* values is narrow and the deviation has little impact on α_1*A*_. Therefore, the value of *θ_A_* is set as 0.21, and then α_1*A*_ = 1.781. $\psi_{0}=\frac{\pi }{4}$ and three angles are put into $I_{L}(\psi_{0})$, $I_{C}(\psi_{0})$, and $I_{S}(\psi_{0})$ to obtain the value of $I_{LA}(\frac{\pi }{4})=1.1168$, $I_{LB}(\frac{\pi }{4})=1.1478$, $I_{CA}(\frac{\pi }{4})=0.6821$, $I_{CB}(\frac{\pi }{4})=0.6931$, $I_{p}(\frac{\pi }{4})=0.022$, $I_{S}(\frac{\pi }{4})=0.8557$, in which $I_{LA}<I_{LB}$; $I_{CA}<I_{CB}$. Thus, it is clear and well demonstrated that the Mode B folded lobe consumes more energy than the folded lobe of Mode A.

Three folding angles are integrated into Equations (19)–(21), and the calculation formulas of energy consumption for each element in the square tube are obtained as follows:

(25) $$E_{L-\mathrm{shape}}=16M_{0}\left[1.781c_{m}+\frac{1.117H^{2}}{b}+\frac{0.704H^{2}}{h}+\frac{3.403Hb}{h}\right],$$

(26) $$E_{T-\mathrm{shape}}=8M_{0}(N-1)\left[5.133c_{m}+\frac{5.656H^{2}}{b}+\frac{2.794H^{2}}{h}+13.691\frac{Hb}{h}\right],$$

(27) $$E_{\mathrm{crisscross}}=8M_{0}{\left(N-1\right)}^{2}\left[1.571c_{m}+\frac{1.148H^{2}}{b}+\frac{0.715H^{2}}{h}+\frac{3.423Hb}{h}\right].$$

The plates in the tube are an orderly connected whole, where the folding heights of the crisscross, T-shaped, and L-shaped elements affect each other. When the cell width is the same, the element heights between them are also the same. According to the law of conservation of energy, the relation between the average force and the total energy consumption of the square tube (*E_totalI_*) is $P_{m}=\frac{E_{total}}{2H}=\frac{E_{L-shape}+E_{T-\mathrm{shape}}+E_{crisscross}}{2H}$, which can be obtained by combining Equations (25)–(27):

(28) $$P_{m}=M_{0}\left(q_{1}\frac{c_{m}}{H}+q_{2}\frac{H}{b}+q_{3}\frac{H}{h}+q_{4}\frac{b}{h}\right),$$

where

$$\begin{array}{l} q_{1}=3.142N^{2}+3.982N;\\ q_{2}=4.592N^{2}+13.44N-9.096;\\ q_{3}=2.86N^{2}+5.456N-2.684;\\ q_{4}=13.691N^{2}+27.308N-13.848 \end{array}.$$

According to the least action principle, the variation in the mean crushing force is taken as 0, that is, for any *δ_H_* and *δ_b_*, the following conditions need to be met:

(29) $$\frac{𝜕P_{m}}{𝜕H}=\frac{𝜕P_{m}}{𝜕b}=0.$$

The total width of the square tube is set to be *C_total_*. *θ* = 0.21 is substituted into Equation (29), and then $\frac{H}{b}=4.4221$. Combined with Equation (28), we can calculate the following:

(30) $$b=\sqrt{\frac{q_{1}hc_{m}}{q_{3}+0.226q_{4}}}=I_{b}(N)\sqrt{\frac{hC_{total}}{2N}},$$

(31) $$H=4.4221I_{b}(N)\sqrt{\frac{hC_{total}}{2N}},$$

(32) $$P_{m}=M_{0}\left(4.4221q_{2}+\left(4.648\sqrt{(q_{3}+0.226q_{4})q_{1}}\right)\sqrt{\frac{C_{total}}{2Nh}}\right),$$

where $I_{b}(N)=\sqrt{\frac{3.142N^{2}+3.982N}{5.954N^{2}+11.628N-5.814}}$; hence, it is evident that the number of cells exerts an influence on the values and of *H* and *b*, as well as the mean crushing force.

## 6. Formula for Peak Initial Crushing Force

### 6.1. Specification of Critical Force

**Figure 17:**
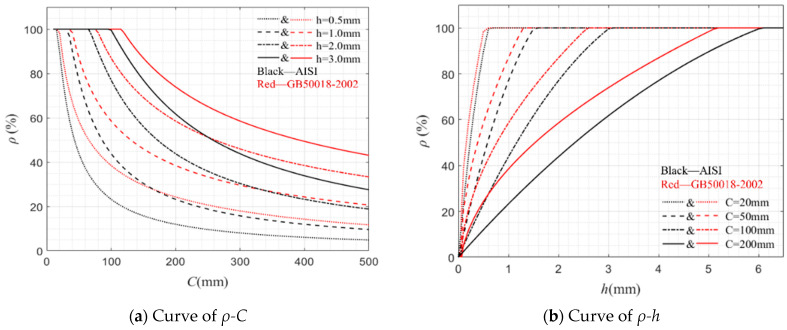
Correlation curves between *ρ* and *C*, *ρ* and *h* in AISI and GB20018-2002.

The initial crushing force can reflect the ultimate bearing capacity of tubes in the critical state, and is equally important for evaluating the crashworthiness. According to the theory of thin plate bending, the expression for the axial critical stress of an independent thin-walled plate can be obtained as follows:

(33) $$\sigma_{cr}=\frac{k\pi^{2}E_{0}}{12(1-\nu^{2})}{\left(\frac{h}{C}\right)}^{2},$$

where *k* is the constraint coefficient at the end or the stability coefficient of the plate after compression, *C* is the width of the thin plate, and *ν* is the Poisson’s ratio of the material. Considering that the stress on the cross section of square tubes subjected to axial compression is not uniform and the stress near the tube corner is large and concentrated, the effective width method in steel structure design in this study is selected as a reference for calculating the critical force of square cross-sectional tubes, so as to simplify the calculation of critical force. In the AISI steel structure design specification [31], the effective width method is used to calculate the ultimate bearing capacity of the axial compression of the structural steel. The calculation formula for the square cross-sectional tube can be written as

(34) $$P_{cr}=\sigma_{y}hb_{e},$$

(35) $$\lambda =\sqrt{\frac{\sigma_{y}}{\sigma_{cr}}},$$

(36) $$\rho =\left\{\begin{array}{ll} 1 & \lambda \leq 0.673\\ (1-\frac{0.22}{\lambda })/\lambda & \lambda >0.673 \end{array}\right.,$$

(37) $$b_{e}=\rho C,$$

where *ρ* is the effective cross-sectional coefficient, *b_e_* refers to the effective width, and *λ* represents the relevant proportional coefficient. The critical force *P_cr_* can be obtained by calculating the product of the cross section and yield stress under effective width. In the Chinese standard “Technical code of cold-formed thin-wall steel structures” (GB50018-2002) [32], a calculation formula for the effective width of the three-section formula is proposed, in which the relationships between the width of the effective section and the width of tubes, as well as the thickness, are specified:

(38) $$b_{e}=\left\{\begin{array}{ll} C & \frac{C}{h}\leq 18\alpha \rho_{c}\\ \left(\sqrt{\frac{21.8\alpha \rho_{c}h}{b}}-0.1\right)C & 18\alpha \rho_{c}<\frac{C}{h}<38\alpha \rho_{c}\\ \frac{25\alpha \rho_{c}C}{C} & \frac{C}{h}\leq 38\alpha \rho_{c} \end{array}\right.,$$

where α is the calculation coefficient of distribution uniformity, and *ρ_c_* represents the coefficient of influence that considers the constraints of the plate group and the compressive stability of the plate, i.e., $\rho_{c}=\sqrt{\frac{205lk}{\sigma_{1}}}$; *σ*_1_ is the reduced stress considering the stability of the component, and *l* is the constraint coefficient of the plate group. In comparison to the specifications in AISI, GB50018-2002 considers more factors and is more detailed regarding the calculation of critical forces for plates. It is worth noting that although critical buckling stress based on elastic assumptions is adopted in both GB50018-2002 and AISI, the constraints of the plate and the uniformity of stress distribution are also considered in GB50018-2002. In addition, the influence of the critical stress *σ_cr_* under compression on the effective width value is weakened by adding the above correction parameters, making the parameter values and calculations tend to be empirical. The correlation curves between *ρ* and *C*, as well as the effective cross-sectional coefficient and *h* obtained from AISI and GB50018-2002, are shown in Figure 17. Obviously, the width reduction calculated through GB50018-2002 is more conservative compared to that of AISI. In summary, there is still an obvious difference in the peak force calculated by the two in the part with width reduction, and this difference gradually amplifies as the width increases.

According to the differences in calculation formulas between AISI and GB50018-2002, the distribution coefficient of stress uniformity of GB50018-2002 is taken into account in this study in AISI’s formula calculation. In addition, the uniformity of tube wall compression is considered and a two-stage formula is used to calculate the stability coefficient *k*. Therefore, the formula for the critical stress and effective width coefficient of the tubes wall can be detailed as follows:

(39) $$\sigma \prime_{cr}=\frac{lk\pi^{2}E_{0}}{12(1-\nu^{2})}{\left(\frac{h}{C}\right)}^{2},$$

(40) $$\rho \prime =\left\{\begin{array}{ll} 1 & \lambda \prime \leq 0.673\\ (1-\frac{0.22}{\lambda \prime })/\lambda \prime & \lambda \prime >0.673 \end{array}\right.,$$

in which $\lambda \prime =\sqrt{\frac{\sigma_{y}}{\sigma \prime_{cr}}}$. In accordance with GB50018-2002, the width and thickness between adjacent plates are set as *C_i_* and *h_i_* (*I* = 1, 2) respectively. The value of *l* and *k* are as follows:

(41) $$l=\left\{\begin{array}{ll} \frac{1}{\sqrt{\xi }} & \xi \leq 1.1\\ 0.11+\frac{0.93}{{\left(\xi -0.05\right)}^{2}} & \xi >1.1 \end{array}\right.,$$

(42) k=7.8−8.15φ+4.35φ2​ 0<φ≤17.8−6.29φ+9.78φ2​ −1≤φ≤0.

where $\xi =\frac{C_{1}}{C_{2}}\sqrt{\frac{k}{k_{c}}}$. For T-shaped and crisscross elements, $\frac{C_{1}}{C_{2}}$ is the maximum value of all plate width ratios, *k_c_* is the compressive stability coefficient of adjacent walls, and the coefficient of uneven axial pressure is $\varphi =\frac{\sigma_{\max}}{\sigma_{\min}}$. The change in thickness and axial force of the plate will affect the stress $\sigma_{\max}$ and $\sigma_{\min}$ at both ends of the wall. According to Equations (38) and (39), it can be seen that in the case of regular tube walls without obvious thickness changes, the influence on the stability coefficient and the constraint coefficient between plates is relatively small. However, for the design of complex cross-sectional tubes, parameters k and l can effectively correct the coefficient of effective width, so that the calculation deviation can be controlled within a reasonable range.

### 6.2. Critical Force in the Axial Direction of Multicellular Thin-Walled Tubes

**Figure 18:**
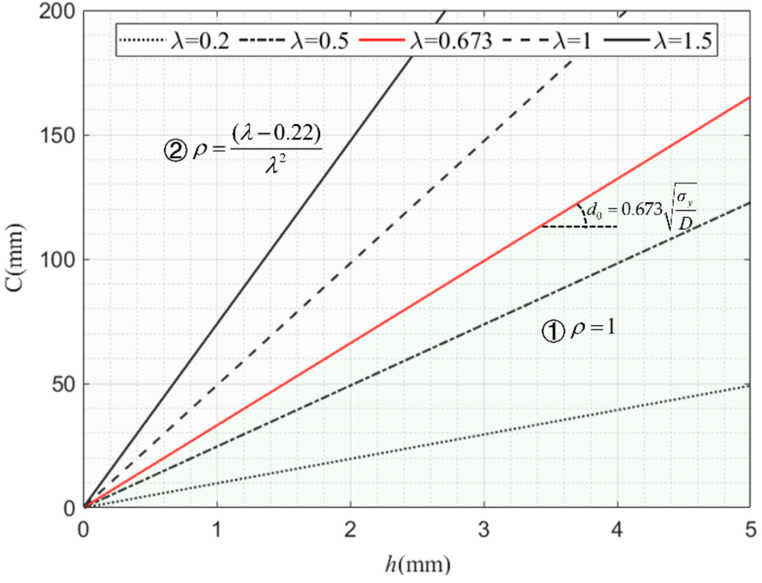
The range of *ρ* with the effects of the *λ* value.

The critical force calculation discussed in Section 6.1 only involves the analysis of the values of single-cell square tube walls. When using AISI specifications for design, changes in the width and thickness of adjacent tube walls can result in inconsistent formulas for critical stress and effective width. Therefore, it is necessary to distinguish between tube walls under different combinations of width and thickness when it comes to design and calculation. Figure 18 shows the relation curve between *C* and *h* under the change in *λ*, in which $D=\frac{lk\pi^{2}E_{0}}{12(1-\nu^{2})}$. The width-to-thickness ratio can be obtained from Equations (32) and (35), that is, $d=\lambda \sqrt{\frac{D}{\sigma_{y}}}$. When *λ* = 0.673, the corresponding width-to-thickness ratio *d*_0_ is the critical ratio of width to thickness. According to the segmented function Equation (35), the set of points is divided (*h*, *C*) into two areas in the *C*-*h* curve: ① and ②, where the method for obtaining the effective width coefficient *ρ* (*h*, *C*) in the two areas is different. Due to the regularity of each lattice form within square multicellular thin-walled tubes, the wall width-to-thickness ratio is the same. Assuming the cell width is $C_{N}=\frac{C_{total}}{N}$, the impact of changing the width of a square cross-sectional tube on the value of *ρ* is discussed in the following parts.

(1) When (*h*, *C_N_*) ∈ Area ①, i.e., $d_{N}\leq d_{0}$, then *ρ_N_* = 1, the effective width *b_eN_* = *C_N_*, and the expression for the critical force can be given as

(43) $$P_{crN}=\sum \sigma_{y}hC_{N}.$$

(2) When (*h*, *C_N_*) ∈ Area ②, i.e., $d_{N}>d_{0}$, and then $\rho_{N}=\frac{\lambda_{N}-0.22}{{\lambda_{N}}^{2}}$, the critical force under axial compression can be given as

(44) $$P_{crN}=\sum \sigma_{y}\rho_{N}hC_{N}=\sum \left(\sqrt{\sigma_{y}D}-\frac{0.22DNh}{C_{total}}\right)h^{2}.$$

From the above, it is clear that the calculation of the axial critical force of a square cross-sectional tube can be attributed to the relationship between the width-to-thickness ratio of the tube wall and the width-to-thickness ratio *d*_0_ of the boundary, in which the calculation process can be simplified through the above derivation. According to the element division and quantitative relationship described in Section 5.2, the number of tube walls can be obtained as n=2N(N+1). Due to the same aspect ratio of lattice walls, the critical force falls into the same region during calculation. Therefore, the critical force of square lattice multicellular tubes is detailed as follows:

(45) $$P_{cr-total}=2N(N+1)\left\{\begin{array}{ll} \frac{\sigma_{y}hC_{total}}{N} & (d_{N}\leq 0.673\sqrt{\frac{D}{\sigma_{y}}})\\ \left(\sqrt{\sigma_{y}D}-\frac{0.22DNh}{C_{total}}\right)h^{2} & (d_{N}>0.673\sqrt{\frac{D}{\sigma_{y}}}) \end{array}\right..$$

## 7. Discussion

### 7.1. Verification of Axial Compression Theory

**Figure 19:**
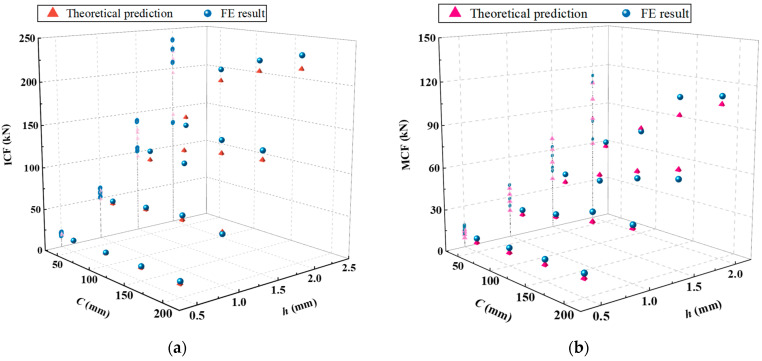
Differences between numerical simulation and theoretical prediction values of single-cell tubes: (**a**) the peak force of crushing distributed with *h* and *c*; (**b**) the average force of crushing distributed with *h* and *c*.

In this section, a comprehensive comparative analysis of the accuracy of energy consumption calculations for single-cell square tubes is made through finite-element simulation. In addition, the finite-element parameter of both the material and finite-element properties outlined in Table 1 and Section 3 is still used to calculate the mean and peak crushing forces, in which the thicknesses of the single-cell square tube wall selected for practical engineering are 0.5 mm, 1.0 mm, 1.5 mm, and 2.0 mm, and the widths are 50 mm, 100 mm, 150 mm, and 200 mm, respectively. Meanwhile, the height of the tube body is set at 300 mm. The specific research results are shown in Table 6, and the comparison between theoretical and finite-element values is shown in Figure 19a,b.

From the data in Table 6 and Figure 19a, it can be seen that when the values of *h* and *C* are small, the predicted *ICF* value obtained through theoretical calculation is smaller than the finite-element simulation value; when the value of *h* increases to the range around 1.5 mm, the theoretical predicted *ICF* value begins to exceed the finite-element simulation value, in which the difference between the theoretical predicted *ICF* value and the finite-element simulation value slightly increases with the increase in the value of *C*. However, the theoretical predicted results are in good agreement with the finite-element simulation results, and the deviation rate between these two is relatively small. In terms of average force, there is also the phenomenon that the theoretically calculated *MCF* is slightly smaller than the finite-element simulation values when the values of *h* and *C* are small. Nevertheless, with gradual increases in the values of *h* and *C*, the projection of numerical points in the *z*-axis direction of the curve shows that the mapping points are sparsely distributed when the value of *h* is large (Figure 19b). Then, the deviation rate between the two shows a fluctuation of positive and negative changes, which is to some extent related to the stability of the tube body during folding. The maximum deviation rate of *MCF* is −32.87%, but the deviation value is only 3.28 kN. By referring to the abundant works in the literature [8,26,33,34], we concluded that, for numerical deviations with small bases, a force within 4 kN is acceptable. In general, the predicted *ICF* values for the axial crushing of single-cell tubes are relatively close to the values simulated by the finite-element analysis, and the error deviation is relatively small but shows a growing trend with increasing thickness.

**Figure 20:**
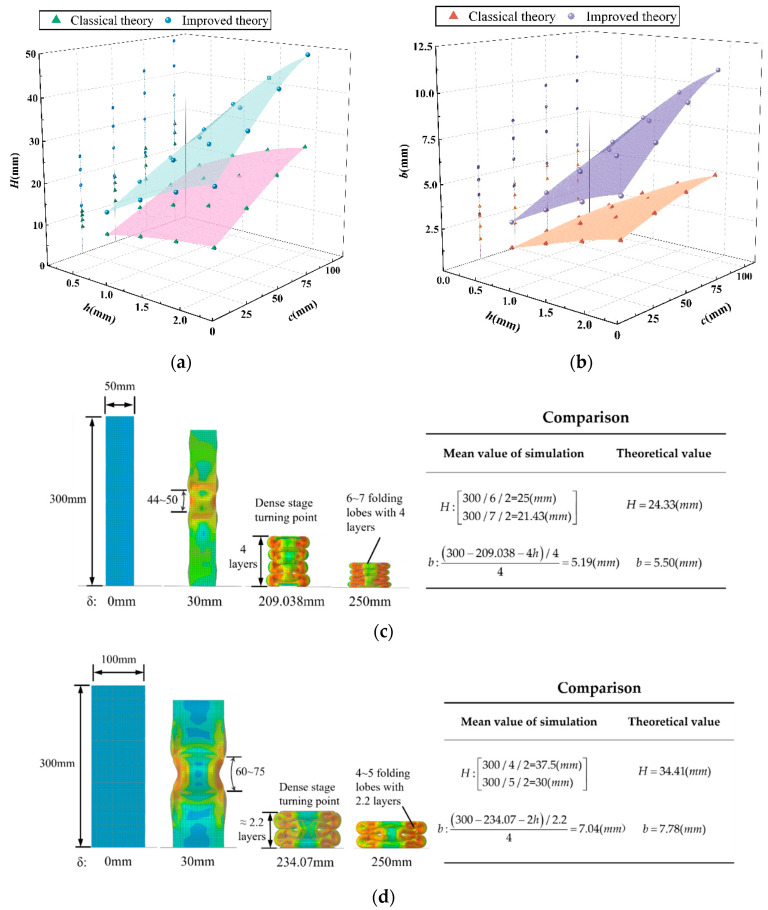

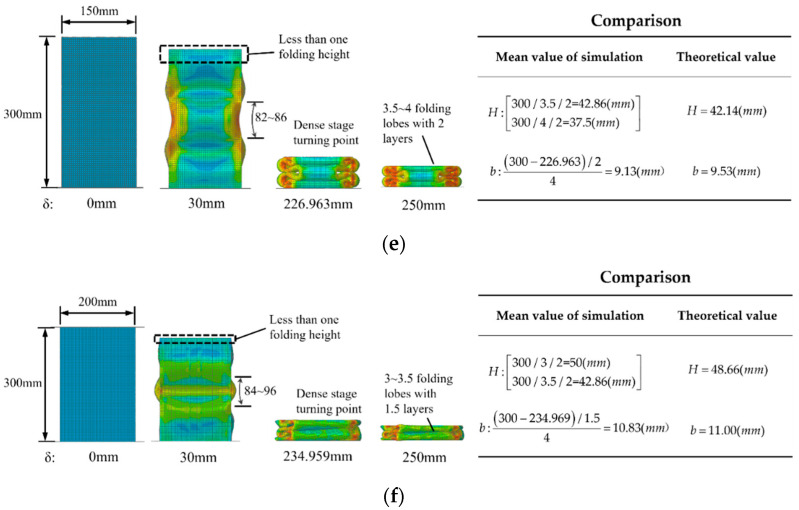
Comparison of theoretical and finite-element values for *H* and *b*: (**a**) curved surface where *H* varies with *h* and *c*; (**b**) curved surface where *b* varies with *h* and *c*; (**c**) FE simulation diagram of specimen S1-4; (**d**) FE simulation diagram of specimen S2-4; (**e**) FE simulation diagram of specimen S3-4; (**f**) simulation of specimen S4-4.

The element height *H* and the bending radius *b* of the folded lobe are key parameters for the calculation of the folding formula. In Section 5.4, the values of *H* and *b* are obtained, and the calculated values are compared with those in the classical super folding element theory, as shown in Figure 20a,b. From the comparison of the fitting surface, it can be seen that the values obtained with the improved theory differ significantly from those obtained with the classical super folding theory. Meanwhile, the theoretical values of convex panel stretching, whether *H* or *b*, are greater than those obtained in the classical theory, and the deviation rate of the predicted values increases with the increase in the values of *h* and *C.* To further verify the accuracy of the theory, the FE models of specimens of S1-4, S2-4, S3-4, and S4-4 were selected as representatives for a qualitative analysis of the simulation results. From Figure 20c–f, it can be found that the range of element heights formed by the four FE models after crushing is [44, 50], [60, 75], [82, 86], and [92, 100], respectively, by calculating the number of grids. Additionally, the theoretical prediction of 2*H* is within the corresponding range mentioned above, which can also be inferred from the total number of folded lobes formed by the tube after folding. By calculating the rate of the remaining height to the number of folded layers at the turning point of the FE simulation in the densification stage, the bending radius of the outer folded lobe under four working conditions can be obtained, with the corresponding values of b being 5.69 mm, 8.24 mm, 9.13 mm, and 10.83 mm, respectively. In accordance with Table 6, the theoretical values of b differ from the FE simulation results by 0.19 mm, 0.46 mm, 0.4 mm, and 0.17 mm, respectively, and the deviation rates are only 3.3%, 5.6%, −4.4%, and −1.6%, respectively, which indicates that the calculated values are basically consistent with the model results. In summary, the theory and model in this study conform to the numerical simulation results in terms of the *MCF* and *ICF*, as well as the parameters *H* and *b*. Therefore, it can be considered that this calculation model is suitable for practical engineering designs.

### 7.2. Optimization of Variables N and h

The single-cell square tube is a special case of multicellular square tube with *N* = 1, which has been verified using the finite-element method in Section 7.1 and is considered to be effectively applicable by folding theory. In engineering, there are often design limitations on the cross-sectional width *C_total_* of this tube, while multicellular square tubes can form a diverse combination by changing the number of segments and wall thickness. In this study, *N* and *h* are set as variables, the optimization method of *N* and *h* under the condition of fixed *C_total_* is discussed, and the height of the tube is uniformly specified as *L* = 300 mm.

#### 7.2.1. Construction of Response Surface

In practical engineering designs, various indicators such as peak pressure, average force, and energy consumption are often taken into account, while *SEA* can simultaneously reflect the changes in *MCF*, tube mass, and energy consumption, which can be regarded as a suitable analysis index [35]. According to Equations (1) and (3), $SEA=\frac{P_{m}\delta_{e}}{m}$ can be derived. In view of geometric relationships, the mass of square lattice multicellular tubes can be expressed as follows:

(46) $$m=A_{N}\rho L=\rho hL(N+1)\left[2C_{total}-(N+1)h\right].$$

**Figure 21:**
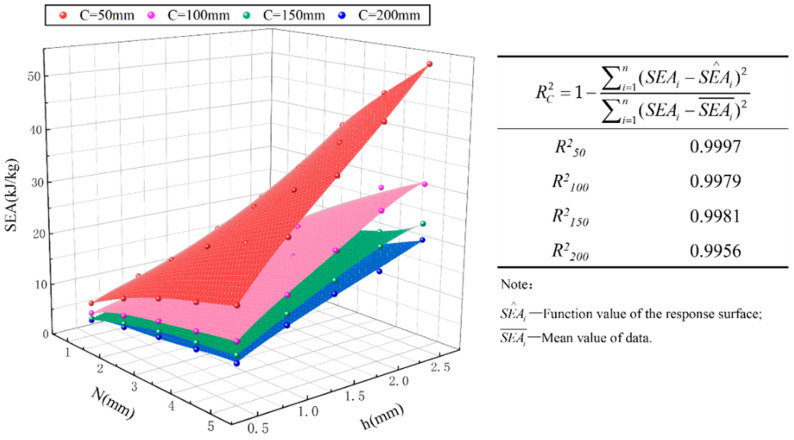
*SEA* response surfaces with different cross-sectional widths.

The effective stroke $\delta_{e}$ is related to *N* and *h*, as well as the cross-sectional width *C_total_.* To obtain the specific energy absorption *SEA* corresponding to different aspect ratios and cell numbers, the *SEA* values obtained by combining the finite-element simulation results of Equations (32) and (46) in this study are shown in Table 7. Xiang [36] summarized the literature and indicated that the effective crushing distance to original length could be in the range of 65% and 80%. According to the results in Table 7, the $\delta_{e}$ values can all meet the above range. Subsequently, the polynomial response surface method (PRSM) is used to construct a proxy model for *SEA* [5]. It has been confirmed that the use of a full fourth-order function as the fitting function can meet the accuracy requirements of *SEA* under bivariate conditions [8,35]. The values of effective stroke $\delta_{e}$ are obtained through finite-element simulation and the response surfaces of *SEA* with *C_total_* of 50 mm, 100 mm, 150 mm, and 200 mm are drawn, as shown in Figure 21. The corresponding fourth-order response surface functions are shown in Appendix A. By calculating the R^2^ (coefficient of determination) of the response surface and finite-element data, it is clear that the fitting degree of the response surface is relatively good, so this function can be used for optimization analysis. On the whole, as the values of *N* and *h* increase, the values of *C_total_* decrease and *SEA* also increases, which is related to the mass changes in the tube body.

#### 7.2.2. Pareto Front

In this study, the critical force *ICF* and specific energy absorption *SEA* are used as optimization indicators for the collision resistance performance of multicellular tubes [37]. From the non-dominated sorting genetic algorithm (NSGAII), a set of Pareto solutions with high accuracy can be obtained through non-dominated sorting while maintaining population diversity, which is suitable for nonlinear optimization of *SEA* and critical forces [38,39]. Given its superiority, NSGAII was selected in this study for multi-objective optimization, so as to determine the objective functions and constraints, which can be detailed as follows:

(47) Minimize​ F1(C,h)​ =ICF(N,h)Maximize​ F2(C,h)=SEA(N,h)s.t​ 1≤N≤50.5​ mm≤h≤2.5​ mm​ .

**Figure 22:**
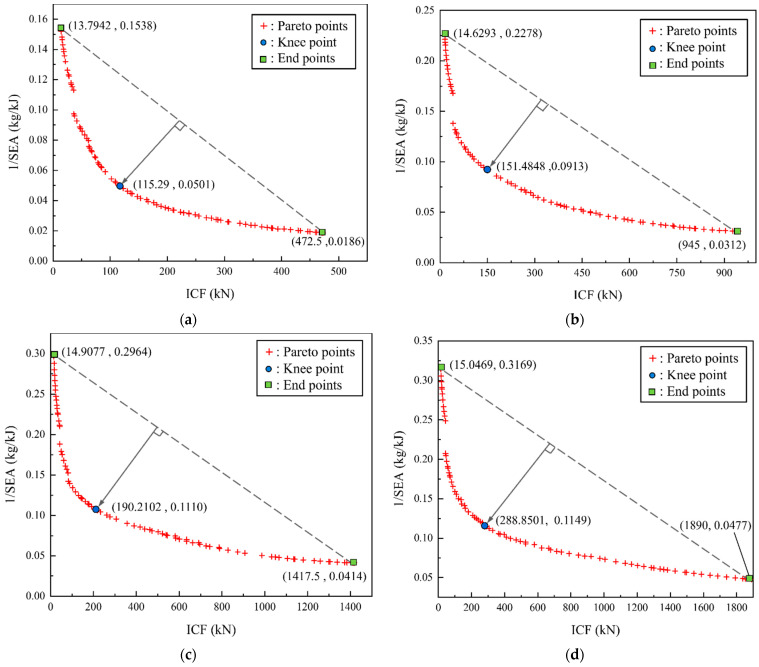
Pareto points under different cross-sectional widths and knee points: (**a**) *C_total_* = 50 mm; (**b**) *C_total_* = 100 mm; (**c**) *C_total_* = 150 mm; (**d**) *C_total_* = 200 mm.

The Pareto frontier values obtained under different cell numbers are shown in Figure 22. The selection of the optimal point of the Pareto solution set is often related to the actual engineering requirements and design direction.

The knee point on the front line represents that the larger value of *SEA* should be selected as much as possible without a sharp increase in *ICF*. The inflection point along the front line is believed to be an ideal solution in the decision space [8,20]. Given that the front line of this study is a convex line with only a single knee point, the identification of ideal Pareto points under multi-objective optimization can be carried out by using the Normal Boundary Intersection Method (*NBI*) for exploration [40,41]. The payoff matrix composed of boundary functions is defined as $P=\left[\begin{array}{cc} ICF_{1}(N,h) & ICF_{2}(N,h)\\ SEA_{1}(N,h) & SEA_{2}(N,h) \end{array}\right]$. The payoff matrices for these four widths set based on the variable range and optimization results are detailed in the following:

(48) $$\begin{array}{ll} P_{50}=\left[\begin{array}{cc} 13.7942 & 472.5\\ 0.1538 & 0.0186 \end{array}\right] & P_{100}=\left[\begin{array}{cc} 14.6293 & 945\\ 0.2278 & 0.0312 \end{array}\right];\\ P_{150}=\left[\begin{array}{cc} 14.9077 & 1417.5\\ 0.2964 & 0.0414 \end{array}\right] & P_{200}=\left[\begin{array}{cc} 15.0469 & 1890\\ 0.3169 & 0.0477 \end{array}\right], \end{array}$$

**Figure 23:**
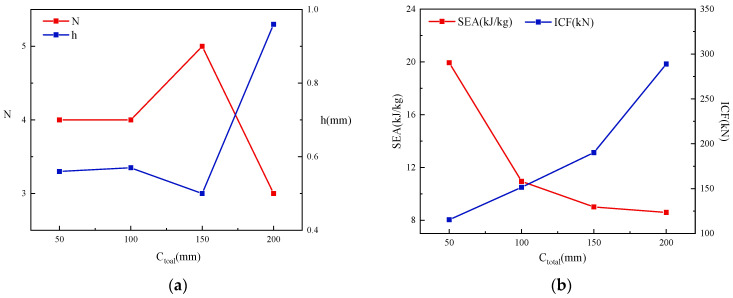
Impact of *C_total_*: (**a**) *C_total_*—*N*-*h*; (**b**) *C_total_*—*SEA*-*ICF*.

The knee points are shown in Figure 22. The curves in Figure 22a–d all present obvious convex shapes, which indicates a conflict between *SEA* and *ICF* with *N* and *h* as optimization variables. Therefore, it is necessary to discuss the optimization of *N* and *h*. Data on the *SEA* and *ICF* of the four knee points are summarized in Table 8. From the crushing force efficiency of the four different cross-sectional widths, it can be found that the *CFE* of the optimal solution for multicellular square tubes is not linearly correlated with *C_total_*. When *C_total_* increases from 50 mm to 150 mm, the *CFE* will increase from 42.75% to 45.00%. However, when *C_total_* = 200 mm, *CFE* will decrease to 43.10%, even less than the *CFE* value at *C_total_* = 100 mm, which suggests that there is a peak value in the *CFE* of cells when *C_total_* changes. From the changes in the values of the optimization variables in Figure 23a, the number of pores in the optimized overall cell remains in the range of 3 × 3 to 5 × 5, indicating that the multicellular form is conducive to improving the strength and energy consumption of the tubes. When the width increases to 200 mm, the number of optimal cells will decrease and the thickness will obviously increase. In Figure 23b, the changes in *SEA* and *ICF* are demonstrated. Generally, *SEA* shows a decreasing trend with the variation in cross-sectional width, while the change in trend of *ICF* is opposite to that of *SEA*, which should be the result of the increasing influence of the multicellular tube’s mass on the optimization of larger cross-sectional width conditions. Comparing Figure 23a,b, it can be inferred that the influence of *N* on mass is greater than that of *h*. To compensate for the decreasing trend of *SEA* due to the impact of mass, a relatively rapid increase in *h* is induced in this study. When *C_total_* increased from 150 mm to 200 mm, *h* increased by 92%.

From the above optimization results, a reasonable value range of *C_total_* is needed to ensure superior crashworthiness and energy consumption. With design permission, *C_total_* can be placed within a range of around 150 mm to obtain a *PF* point with higher *SEA* and lower *ICF*. Additionally, it is worth noting that the *PF* knee point selected in this study is the optimal balance point in the absence of special requirements for dual indicators. In practical engineering designs, the allowable design range for *SEA* and *ICF* can be specified according to actual requirements, so that the intervals of variables of *N* and *h* can be determined in *PF*. In this way, on the premise of determining the width of the multicellular tubes, the optimal combination of *N* and *h* required by the index can be clearly obtained from the optimization results. However, the previous optimization of the tube often focuses on the optimal state of the tube width and wall thickness under the exact number of cell pores, which is also the reference of the formula and optimization method proposed in this paper.

## 8. Conclusions

In order to study the calculation methods for the average force and peak crushing force of lattice-filled square multicellular cross-sectional tubes, the super folding element theoretical model and the calculation formula for peak crushing force are summarized and improved in this study based on the force calculation of single-cell square cross-sectional tubes. Additionally, the calculation method is extended from single-cell square tubes to thin-walled square multicellular tubes. Furthermore, in accordance with cross-sectional and deformation characteristics, elements with the same compression forms are classified in this study, for the purpose of obtaining the calculation formulas for the peak force and average force of multicellular tubes. In addition, a response surface is constructed, and a multi-objective algorithm is used to optimize the square lattice multicellular tubes with dual indicators. The specific conclusions of this study are as follows:

(1) A super folding element model that considers convex panel stretching is proposed based on the classic super folding element model and the extended folding element model. Meanwhile, the folded-lobe model and double-hinged-line model are introduced, and the corresponding reference planes are set. Moreover, the partition problem of the calculation of the cross section of energy consumption’s area has been solved, making the calculation of energy consumption of the tube more convenient.
(2) A comparison and analysis of the critical crushing force calculation formulas in AISI and GB50018-2002 specifications is made, which takes into consideration the stability coefficient of the plate group in the effective width calculation. Meanwhile, the critical force formulas based on the influence of the width-to-thickness ratio are summarized. In the expansion of multicellular tubes, the width-to-thickness ratio of cells and the number of cells are taken as parameters so as to obtain the calculation formula for multicellular tubes.
(3) Additionally, the folding element in square lattice multicellular cross-sectional tubes is divided into the following types based on the folding characteristics: L-shaped element, T-shaped element, and crisscross element. According to the differences in the folded-lobe model, the folding element is divided into Mode A and Mode B. Ultimately, the average collapse response value of multicellular tubes is calculated for the participation of five energy-consuming regions in the three element types.
(4) Based on the NSGAII method, *SEA* and *ICF* are optimized by regarding *N* and *h* as variables, and the *NBI* method is used to obtain knee points, aiming to explore the influence of *N* and *h* on the optimized square lattice multicellular tubes under different *C_total_* values. Finally, optimization directions and references are provided in this study for future research.

In conclusion, the lattice-filled multicellular square tube structure is characterized as exhibiting superior performance for the obvious buffering capability and energy absorption. By proposing the two reference planes, improving the super folding element, and expanding the effective section method, the calculation method and formula proposed in this study for the average crushing force and critical peak force of multicellular square sections are relatively reasonable. Therefore, the lattice-filled multicellular square tube can not only be used for engineering designs and crashworthiness analyses, but can also provide novel perspectives for optimizing the design of the cross section of square lattice multicellular tubes.

## Figures and Tables

**Table 1:** Material parameters of the square tubes.

Material	Young’s Modulus *E*_0_ (MPa)	Yield Strength *σ*_0.2_ (MPa)	Ultimate Strength *σ*_u_ (MPa)	Hardening Coefficient *n*	Poisson’s Ratio ν	Density (kg/mm^3^)
304 Austenitic Stainless Steel	210,000	315	632.58	6	0.3	7.93 × 10^−6^

**Table 2:** Comparison of crushing force and average force.

Specimen Number	Dimensions of Square Tube cross Section (mm × mm)	Thickness of Tube Surface (mm)	*ICF* (kN)			*MCF* (kN)		
			Value of the Experiment	FE Analysis	Error	Value of the Experiment	FE Analysis	Error
T1	50 × 50	1.5	101.65	96.01	5.54%	40.22	39.86	0.90%
T2	50 × 50	2	128.32	122.49	4.54%	54.58	52.74	3.37%
T3	100 × 100	1.5	113.89	107.30	5.83%	46.16	44.61	3.35%
T4	100 × 100	2	195.79	186.87	4.56%	81.60	80.53	1.31%

**Table 3:** Reference plane for the calculation of folded areas.

Energy Consumption Zone of Folding Element	Reference Plane I	Reference Plane II
Area of inclined traveling hinge	√	—
Area of horizontal fixed hinge	√	—
Area of conical surface stretching	√	—
Area of convex panel stretching	—	√
Area of the toroidal shell surface stretching	—	√

**Table 4:** Number of energy consumption zones for the L-shaped, T-shaped, and crisscross elements.

Type	Number of Zones				
	Horizontal Fixed Hinge Line *m_H_*	Oblique Traveling Hinge Line *m_L_*	Stretch Zone of Conical Surface *m_C_*	Stretch Zone of Convex Panel *m_P_*	Toroidal Shell Surface *m_S_*
L-shaped element	2	2	1	1	1
T-shaped element	3	2	2	1	2
Cross-shaped element	4	4	2	2	2

**Table 5:** Total number of models in *N* × *N* square cell tubes.

Type	Folded-Lobe Model		Double-Hinged-Line Model
	Type A	Type B	
L-shaped element	8	0	4
T-shaped element	8(N−1)	4(N−1)	4(N−1)
Crisscross-shaped element	0	$4{\left(N-1\right)}^{2}$	$2{\left(N-1\right)}^{2}$

**Table 6:** Theoretical and finite-element simulation results of single-cell square tubes.

Number	Dimensions of Square Tube cross Section (mm × mm)	Thickness of Tube Surface (mm)	*ICF* (kN)			*MCF* (kN)			Theoretical Calculation of *H* (mm)	Theoretical Calculation of *b* (mm)
			Theory	FE	Diff (%)	Theory	FE	Diff (%)		
S1-1	50 × 50	0.5	13.77	13.81	−0.11	6.70	9.98	−32.87	12.16	2.75
S1-2	50 × 50	1.0	48.50	51.55	−7.72	19.87	23.54	−15.59	17.20	3.89
S1-3	50 × 50	1.5	94.33	105.09	−10.24	37.79	44.83	−15.70	21.07	4.76
S1-4	50 × 50	2.0	126.54	130.64	−3.14	59.84	66.62	−10.18	24.33	5.50
S2-1	100 × 100	0.5	14.59	15.48	−5.75	9.16	12.52	−26.84	17.20	3.89
S2-2	100 × 100	1.0	55.08	57.63	−4.43	26.81	29.64	−9.55	24.33	5.50
S2-3	100 × 100	1.5	116.53	101.4	14.92	50.54	46.48	8.73	29.80	6.74
S2-4	100 × 100	2.0	194.01	207.7	−6.59	79.48	75.54	5.22	34.41	7.78
S3-1	150 × 150	0.5	14.87	16.59	−10.37	11.04	15.12	−26.98	21.07	4.76
S3-2	150 × 150	1.0	57.27	62.67	−8.62	32.14	38.45	−16.41	29.80	6.74
S3-3	150 × 150	1.5	123.93	139.64	−11.25	60.32	55.61	8.47	36.49	8.25
S3-4	150 × 150	2.0	211.55	224.33	−5.70	94.54	110.61	−14.53	42.14	9.53
S4-1	200 × 200	0.5	15.00	17.84	−15.92	12.63	16.21	−22.09	24.33	5.50
S4-2	200 × 200	1.0	58.37	56.98	2.44	36.63	39.59	−7.48	34.41	7.78
S4-3	200 × 200	1.5	127.63	138.51	−7.86	68.57	61.66	11.21	42.14	9.53
S4-4	200 × 200	2.0	220.32	235.97	−6.63	107.24	112.44	−4.62	48.66	11.00

**Table 7:** *δe* and *SEA* values under axial crushing.

Number of Cells *N* × *N*	*h* (mm)	Section Width *C_total_* (mm)							
		50		100		150		200	
		*δ_e_* (mm)	*SEA* (kJ/kg)	*δ_e_* (mm)	*SEA* (kJ/kg)	*δ_e_* (mm)	*SEA* (kJ/kg)	*δ_e_* (mm)	*SEA* (kJ/kg)
1 × 1	0.5	227.98	6.49	233.76	4.52	219.77	3.41	230.98	3.07
2 × 2	0.5	219.46	10.28	223.16	6.92	220.69	5.43	224.46	4.70
3 × 3	0.5	219.65	13.12	227.56	8.88	223.94	6.89	225.65	5.89
4 × 4	0.5	216.92	15.33	220.22	10.07	223.63	8.02	223.92	6.79
5 × 5	0.5	218.63	17.62	220.17	11.38	222.03	8.97	221.21	7.53
1 × 1	1	229.76	9.79	227.98	6.49	213.14	4.83	233.76	4.52
2 × 2	1	216.16	15.69	219.46	10.28	222.88	8.18	234.14	7.27
3 × 3	1	219.56	20.72	219.65	13.12	220.24	10.23	227.56	8.88
4 × 4	1	210.22	23.80	216.92	15.33	215.48	11.76	220.22	10.07
5 × 5	1	209.17	27.34	213.21	17.18	222.97	13.80	227.43	11.75
1 × 1	1.5	211.77	11.56	210.14	7.55	227.98	6.49	217.77	5.27
2 × 2	1.5	208.69	19.91	218.88	13.20	227.51	10.66	217.69	8.57
3 × 3	1.5	209.94	26.41	215.24	16.72	219.65	13.12	231.81	11.57
4 × 4	1.5	207.63	31.71	209.48	19.40	210.99	14.91	219.63	12.91
5 × 5	1.5	204.03	36.35	216.97	23.07	218.63	17.62	226.69	15.13
1 × 1	2	221.98	14.54	229.76	9.79	215.77	7.24	203.97	5.80
2 × 2	2	209.46	24.49	228.14	16.56	214.69	12.02	235.56	11.03
3 × 3	2	207.65	32.42	219.56	20.72	228.81	16.45	219.65	13.12
4 × 4	2	202.92	38.87	210.22	23.80	215.63	18.44	228.79	16.17
5 × 5	2	197.21	44.48	217.43	28.42	222.69	21.81	213.21	17.18
1 × 1	2.5	211.19	16.01	220.41	10.75	204.77	7.83	245.79	7.95
2 × 2	2.5	216.90	29.91	218.08	18.36	235.36	15.18	226.90	12.20
3 × 3	2.5	211.32	39.35	207.95	22.93	218.82	18.24	208.58	14.38
4 × 4	2.5	203.80	47.02	217.59	28.97	203.56	20.27	218.80	17.91
5 × 5	2.5	195.74	53.67	205.04	31.69	211.45	24.22	225.64	21.14

**Table 8:** Dual-indicator optimization results under the application of the NBI method.

Section Width *C_total_*	Optimal Parameters Based on *PF* Knee Points		*SEA* (kJ/kg)	*ICF* (kN)	*CFE*
	*N*	*h* (mm)			
50	4	0.56	19.94	115.29	42.75%
100	4	0.57	10.95	151.48	44.70%
150	5	0.5	9.01	190.21	45.00%
200	3	0.96	8.59	288.85	43.10%

## Data Availability

Data are contained within the article.

## Appendix A

The form of the full fourth-order function under the dual variables *N* and *h* is

$$\begin{array}{ll} F(N,h)= & z_{0}+A_{1}N+A_{2}h+B_{1}N^{2}+B_{2}Nh+B_{3}h^{2}+C_{1}N^{3}+C_{2}N^{2}h+C_{3}Nh^{2}+C_{4}h^{3}\\ & +D_{1}N^{4}+D_{2}N^{3}h+D_{3}N^{2}h^{2}+D_{4}Nh^{3}+D_{5}h^{4} \end{array}$$

Equations for *SEA* response surface under different cell numbers:

In the case of *C_total_* = 50 mm:

$$\begin{array}{ll} \mathrm{SEA}(N,h)= & -0.8698+1.23739N+10.12792h\\ & +0.38652N^{2}+6.67592Nh-10.13095h^{2}\\ & -0.17358N^{3}-0.76068N^{2}h-0.2258Nh^{2}+3.78533h^{3}\\ & +0.01608N^{4}+0.07783N^{3}h-0.11694N^{2}h^{2}+0.20133Nh^{3}-0.54933h^{4} \end{array}$$

In the case of *C_total_* = 100 mm:

$$\begin{array}{ll} \mathrm{SEA}(N,h)= & -7.1724+8.49628N+17.38888h\\ & -3.71369N^{2}+2.77493Nh-20.24176h^{2}\\ & +0.73867N^{3}-0.26206N^{2}h+0.33588Nh^{2}+9.844h^{3}\\ & -0.05417N^{4}+0.03133N^{3}h-0.06184N^{2}h^{2}-0.03467Nh^{3}-1.68667h^{4} \end{array}$$

In the case of *C_total_* = 150 mm:

$$\begin{array}{ll} \mathrm{SEA}(N,h)= & 0.8128-3.73201N+8.46379h\\ & +3.07969N^{2}+4.11148Nh-9.88429h^{2}\\ & -0.70883N^{3}-1.61308N^{2}h+1.59241Nh^{2}+3.63467h^{3}\\ & +0.05267N^{4}+0.193N^{3}h-0.10245N^{2}h^{2}-0.26133Nh^{3}-0.504h^{4} \end{array}$$

In the case of *C_total_* = 200 mm:

$$\begin{array}{ll} \mathrm{SEA}(N,h)= & -1.547+5.27274N-0.39267h\\ & -3.44568N^{2}+7.19994Nh-2.258h^{2}\\ & +0.84808N^{3}-1.14285N^{2}h-1.63127Nh^{2}+1.29067h^{3}\\ & -0.0675N^{4}+0.04083N^{3}h+0.24816N^{2}h^{2}-0.01333Nh^{3}-0.12h^{4} \end{array}$$
